# A novel PNIPAM-Modified polyurethane/carboxymethyl cellulose photo-thermoresponsive hydrogel loaded with gemcitabine to suppress esophageal cancer cells via VEGF-mediated angiogenic pathway inhibition

**DOI:** 10.1186/s13036-025-00530-y

**Published:** 2025-07-23

**Authors:** Shirong Wu, Huachuan Zhang, Yu Zhang, Ya Zhao, Mengqi Xiang, Liqiong Hao, Jing Chen

**Affiliations:** 1 Department of Medical Oncology, Sichuan Clinical Research Center for Cancer, Sichuan Cancer Hospital & Institute, Sichuan Cancer Center, Affiliated Cancer Hospital of University of Electronic Science and Technology of China, Chengdu, 610041 China; 2 Department of Thoracic Surgery, Sichuan Clinical Research Center for Cancer, Sichuan Cancer Hospital & Institute, Sichuan Cancer Center, Affiliated Cancer Hospital of University of Electronic Science and Technology of China, Chengdu, 610041 China; 3 Department of Nursing care, Sichuan Clinical Research Center for Cancer, Sichuan Cancer Hospital & Institute, Sichuan Cancer Center, Affiliated Cancer Hospital of University of Electronic Science and Technology of China, Chengdu, China; 4 Department of Medical Oncology, Sichuan Cancer Hospital, Medical School of University of Electronic Science and Technology of China, No.55, Section 4, South Renmin Road, Chengdu, 610041 China

**Keywords:** Carboxymethyl cellulose, PNIPAM, Eesophageal cancer, Apoptosis

## Abstract

This study reports the development of a thermoresponsive hydrogel composed of poly(N-isopropylacrylamide)-modified polyurethane (PNIPAM-PU) and carboxymethyl cellulose (CMC) for the controlled delivery of gemcitabine (GEM) in esophageal cancer treatment. Characterization by XRD and FTIR confirmed successful synthesis and structural integrity. The drug loading capacities were 76.2 ± 2.5% for PNIPAM-PU/CMC@GEM and 83.4 ± 3.4% for PNIPAM-PU/CMC@GEM + NIR. SEM analysis revealed pore diameters of 46.96 ± 12.94 μm (PU/CMC) and 42.96 ± 13.49 μm (PNIPAM-PU/CMC@GEM). Rheological analysis showed a sharp sol–gel transition at 37 °C. Drug release reached 25% over 24 h without NIR, increasing to 75% with NIR exposure. In vitro MTT assay showed significant cytotoxicity in KYSE-140 cells, with IC₅₀ values of 52.85 ± 2.5, 90.97 ± 3.6, 60.76 ± 2.8 and 41.59 ± 2.4 µg/mL for GEM, PU/CMC, PNIPAM-PU/CMC@GEM, and PNIPAM-PU/CMC@GEM + NIR, respectively. Biocompatibility assessment using the normal human esophageal epithelial cell line Het-1 A demonstrated increased cell viability (85%) without showing any morphological changes when treated with fabricated hydrogel formulations, confirming the non-toxic nature of the carrier system. Further, JC-1 and AO-EB staining confirmed mitochondrial depolarization and apoptosis of KYSE-140 cells after exposed to PNIPAM-PU/CMC@GEM + NIR. In vivo studies demonstrated tumor inhibition rates of 48.72%, 10.26%, 61.54%, and 74.36% for GEM, PU/CMC, PNIPAM-PU/CMC@GEM, and PNIPAM-PU/CMC@GEM + NIR, respectively, with minimal side effects. qRT-PCR and Western blot analysis showed VEGF downregulation and angiogenesis inhibition. These findings support the potential of PNIPAM-PU/CMC@GEM hydrogel as an effective localized therapy for esophageal squamous cell carcinoma (ESCC).

**Supplementary Information:**

The online version contains supplementary material available at 10.1186/s13036-025-00530-y.

## Introduction

Esophageal squamous cell carcinoma (ESCC) the predominant subtype of esophageal cancer (EC) ranks as the eighth most prevalent cancer and the sixth leading cause of cancer-related mortality globally [1]. ESCC constitutes 90% of esophageal cancer cases in China [2]. Despite the advancements in diagnostic and therapeutic strategies, ESCC remains a formidable challenge in clinical oncology due to its aggressive nature, early metastasis, and poor overall prognosis [3]. The standard therapeutic approach primarily involves chemotherapy, radiotherapy and surgical resection. However, the five year-survival rates of ESCC patients remains dismal, ranging from 15 to 25%, primarily due to high recurrence rates and treatment resistance [4]. Tumor cells frequently acquire multidrug resistance (MDR) following an initial response to chemotherapy, resulting in tumor recurrence [5, 6]. Chemotherapy has demonstrated the capacity to dramatically impede local tumor growth and diminish tumor burden, thereby markedly enhancing the 5-year survival rate of cancer patients [7]. Although initial responses to chemotherapy may be promising, most patients eventually develop resistance, leading to tumor recurrence and progression [8]. The debilitating side effects of chemotherapeutic agents have required the implementation of tailored medication delivery in cancer treatment.

For the past few decades, many different kinds of polymers can combine to generate hydrogels, and these have many different applications owing to their diverse biological properties [9] includes antimicrobial activity [10, 11], promotion of cell adhesion [12], and proliferation [13] and physicochemical properties of the resulting materials [14, 15]. Particularly for biological applications, the diversity of polymeric hydrogels offers a number of potential benefits [16–18]. Products derived from hyaluronate, alginate, starch, gelatin, cellulose, pectin and agarose were utilized for biomedical applications, since they are in the nature of hydrophobicity, biodegradability, and compatibility with cells [19]. Of these, one of the most common components is cellulose which includes, hydroxyl cellulose, carboxymethyl cellulose, hydroxypropyl cellulose, and hydroxypropyl methyl cellulose [20]. In particular, carboxymethyl cellulose (CMC), a hydrophilic cellulose derivative, is made by substituting hydroxyl groups at positions 2, 3 of the cellulose molecules with carboxymethyl groups and had wide applications in the field of tissue engineering, wound healing and drug delivery [21]. Though, CMC has a numerous application in biomedical field, their low mechanical strength restricts its potential uses in many fields [22]. Thus, to enhance the properties of hydrogels, various strategies can be employed such as forming double networks, making composites of hybrids and reinforcing hydrogels with fibres.

Consequently, thermoresponsive hydrogels have been utilized to enhance the accumulation and retention of medication release at the tumor site [23]. Notwithstanding their efficiency, a limited number of thermoresponsive hydrogel-based pharmaceuticals have progressed to clinical trials, and an even smaller fraction has attained FDA approval for oncological therapy [24, 25]. Debilitating side effects such as cardiotoxicity, gastrotoxicity, nephrotoxicity, immunosuppression and myelosuppression can be caused by the use of intravenously injected chemotherapeutic agents, thermoresponsive hydrogels can inhibit systemic circulation [24, 26]. Natural polymers, proteins/polypeptides, PNIPAM, and PEG based block copolymers are some examples of thermoresponsive hydrogels utilized in cancer treatment [27–29]. Of these, PNIPAM may undergo a reversible phase shift due to temperature variations and is utilized in the formation of thermoresponsive hydrogels. PNIAPM possess a lower critical solution temperature (LCST) of 32 ºC, at which it experiences a change from a coil to globule state [30]. The phase transition due to temperature variations is fast and reversible. PNIPAM possesses both a hydrophobic and hydrophilic amide group.

Polyurethanes (PUs) are a significant class of polymers extensively utilized in medical pharmaceuticals, and biomaterials engineering [31]. For synthetic biomaterials, PUs has a number of desirable qualities. They are easily replicable as pure materials, may be shaped into any shape without degradation or negative changes, and can be sterilized without alterations to their form or properties [32]. The biological environment does not negatively impact the physical, chemical, or mechanical qualities of PUs unless these materials were intentionally engineered to be biodegradable. PUs is not associated with any adverse effects such as cellular deterioration or aging, hypersensitivity, allergies, or cancer causing mutagenic, teratogenic, or toxic reactions [33].

**Fig. 1:**
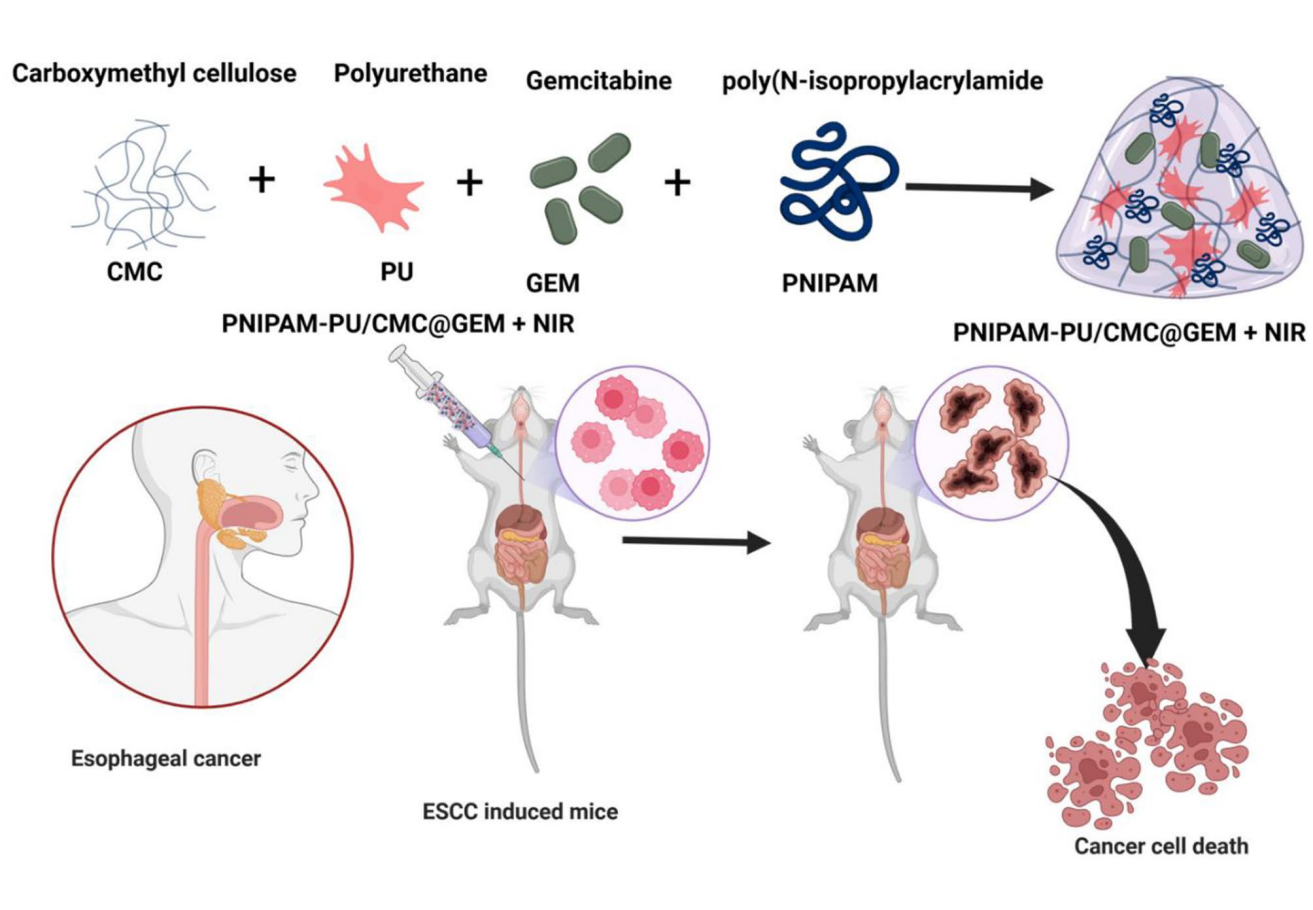
Scheme illustrates the anticancer potential of NIR induced PNIPAM-PU/CMC@GEM hydrogel

Gemcitabine, a nucleoside analogue of cytidine, is effective against various solid tumors, including pancreatic, non-small cell lung, breast, and ovarian cancers, either as a monotherapy or in conjuction with other treatments [34]. However, the systemic administration of GEM is hindered by challenges such as drug resistance and adverse effects linked to high dosage levels [35]. Based on the above-mentioned facts, the current study aimed to develop a thermoresponsive PNIPAM-PU/CMC hydrogel loaded with standard anticancer drug gemcitabine (GEM) and induced with NIR irradiation for the effective and targeted drug delivery to mitigate the viability of esophageal squamous cancer cells (KYSE-140 cells) through in vitro and in vivo analysis (Fig. 1).

## Materials and methods

### Materials, chemicals and animals

Carboxymethyl cellulose (CMC-0.77; average molecular weight 250 kDa, viscosity 735 cps, 2% in H_2_O) (75–85% deacetylated) was purchased form Sigma-Aldrich, USA and used without further purification. Poly (N-isopropylacrylamide) (PNIPAM) was purchased form Acros organics, USA. Polycaprolactone diol (PCL-diol), molecular weight Mn = 2000gmol^-1^. 1, 6 Hexamethylene diisocyanate (HMDI), the catalyst dibutylin dilaurate (DBTDL), and the solvent dimethyl formamide (DMF) were procured from Merck, Germany. Propidium iodide (PI) and 3–4,5-dimethyl-thaizol-2-yl)-2,5-diphenyl tetrazolium bromide (MTT) were procured from Sigma-Aldrich, Shanghai, China. All other chemicals, media and cell culture components used in this study were procured from Invitrogen (Karlsruhe, Company) as analytical grade.

The cell lines (KYSE-140) and Het-1 A were procured from Cell Bank of the Chinese Academy Sciences. In our laboratory, the cell lines were subcultured in RPMI1640 medium at the density of 1 × 10^4^ cells that was supplemented with 10% FBS and 1% Penicillin-streptomycin (ThermoFisher Scientific, Grand Island, USA). The cells were maintained in a humidified chamber at 37 ºC in 5% CO_2_ incubator.

As per the guidelines given by Institutional Animal Care and Use Committee (IACUC) and ARRIVE guidelines, the animal experiment was followed. According to the guidelines given by ARRIVE, a group of healthy 8-weeks-old albino mice of both sexes weight ranges from 25 ± 2 g, were chosen for the experiment. Before start the experiment, the animals were housed in normal polypropylene cages with 6 mice per cage and fed a standard chow diet. In a controlled, pathogen-free environment, the following parameters were maintained to aid acclimatization; 24–28 ºC with 50–60% humidity; 12 h of light and 12 h of dark cycle. The animals were closely monitored for any changes in behaviour, weight loss, or other symptoms related to illness or suffering. Prior to start the experiment, every animal was given with sufficient water and fasted the night. Animals displaying any sign of disease, a typical physiological state or any health complications were excluded from the experiment.

### Synthesis of polyurethane (PU) and fabrication of PU/CMC hydrogel

To make a CMC solution, 2.0 g of sodium carboxymethyl cellulose powder was mixed with 100 mL of distilled water and stirred at room temperature until completely dissolved [36]. As a cross-linking agent, citric acid was added to the CMC and stirred after dissolution at 10% concentration for 20 min before homogenization finalized. After that, 10 mL of the solutions were transferred into plastic petri dishes with the size of 60 mm diameter and allowed to dry at 40 ± 2 ºC for 24 h in order to extract the water. For the crosslinking reaction, we used the slow evaporation approach and maintained the sample at 80 ± 2 ºC for 24 h.

There are two steps to the synthesis of polyurethane (PU). In a three-neck round bottom flask, the isocyanate-terminated prepolymer, PCL-diol, and HMDI are heated at 70 ºC for 2 h during the initial stage of synthesis. The molar ratio of PCL-idol to HMDI utilized is 1:1.08. In the second phase, a chain extension reaction is carried out by adding CMC to the prepolymer in different concentrations while a DBTDL catalyst and DMF are present. The chain extension reaction is finished by adding this and then stirring quickly at 70 ºC for 24 h. Dilution in distilled water caused the chain-extended copolymer that was produced to precipitate. After filtering, the unreacted CMC and diisocyanate from the chain-extended copolymers are successfully rinsed away with distilled water. Further, 20 mg of produced PU was dissolved in 1 mL DMSO and kept it in a stirrer overnight. For making hydrogel, PU/CMC was dissolved in water. This forms PU/CMC hydrogel.

### Preparation of photo-thermoresponsive PNIPAM-PU/CMC hydrogel

For the fabrication of photo-thermoresponsive PNIPAM-PU/CMC hydrogel free radical polymerization method was utilized. In brief, 1.06 mmol of PNIPAM was dissolved in n-hexane at 60 ºC without stirring. Subsequently, the remaining n-hexane was filtered by vaccum filter and kept at -20 ºC for 24 h. After that, 2.0 g of PU/CMC was dissolved in 50 ml of distilled water (pH 10) and mixed with PNIPAM. Then, to ensure the complete dissolution, the mixture was agitated at 90 ºC for 2 h. After cooling to 30 ºC, the mixture was agitated gently for 30 min and 0.976 mL of tetramethyl ethylenediamine (TEMED) was added while being held under nitrogen protection. Afterwards, PU/CMC and the polymer PNIPAM (10 g) were dissolved in distilled water simultaneously. After that, 97.6 mg of ammonium per sulfate was added and allow it to copolymerize under fluorescent light for 24 h at 37 ºC.

For IR-820 loading, 170 mg of IR-820 dye was dissolved in DMSO and mixed thoroughly with 1 mg of the fabricated PNIPAM-PU/CMC [37]. The hydrogel was dispersed in acetonitrile, and the final volume was adjusted to 1 mL. The mixture was stirred gently to allow physical adsorption if IR-820 onto the hydrogel matrix. After that, the IR-820 loaded hydrogel was then purified using 10 kDa Amicon centrifugal filters at 3500 rpm for 10 min followed by three washes with PBS to remove unbound dye. Subsequently, the purified IR-82 loaded PNIPAM-PU/CMC@GEM hydrogel was dispersed in PBS at a concentration of 1 mg/mL and stored at 4 ºC until further use.

### Conjugation of GEM

To load GEM onto PNIAPM-PU/CMC hydrogel, 2.0 mg /mL was dissolved in DMSO. Then 10 mL (0.1 mg/mL) PNIPAM-PU/CMC and 20 µL of triethylamine were added dropwise and kept it in a stirrer. To this mixture, 6 ml of PBS with the pH of 7.4 and the mixture was stirred at room temperature until the GEM loading process was balanced. Afterwards, the GEM loaded PNIPAM-PU/CMC was spun at 20, 000 rpm for 20 min to draw out the components, and distilled water was used for several rinsings. We measured the characteristic absorption peak of GEM at 480 nm to quantify the unbound GEM from the supernatant.

### Characterization of fabricated PNIPAM-PU/CMC hydrogel

Fabricated PNIPAM-PU/CMC@GEM hydrogel was physiochemically characterized utilizing a number of methods. UV-Vis and fluorescence emission spectra analysis was employed to assess the optical properties of the fabricated hydrogel after loaded with IR-820 was recorded using a multimode microplate reader (Varioskan™ Flash, Thermo Fisher Scientific, USA). The structural analysis was determined by employing X-ray diffraction, which is done using a 1.54 Å wavelength Cu Ka source and graphite monochromator on Rigaku small Flex Advance wide-angle X-ray diffractometer. The voltage and current parameters of the generator are kept at 40 kV and 20 mA, respectively. Afterwards, the samples were analysed at a speed of 2º/min. FTIR analysis was carried out to determine the functional groups of hydrogels. For the analysis, the sample was mixed with analytical grade KBr and scanned from the ranges of 400–4000 cm^− 1^. Scanning electron microscopy (JEOL JSM-6490LA) was utilized to study the structural morphology of fabricated hydrogels. Dynamic light scattering (DLS: Brookhaven Instruments Corp., USA) analysis was performed to find out the comprehension properties of the hydrogel.

### Swelling behaviour

We used a gravimetric method to find out the swelling behavior of fabricated hydrogels. The fabricated PU/CMC, PNIAPM-PU/CMC@GEM were kept in a water bath for 15 min. The samples were submerged in PBS (pH 7.4) at 37 ºC after their dry weight (Wdry) was recorded. The swelling weight (Wswollen) was measured by removing the hydrogels at predetermined intervals, blotting them to remove excess water, and then weighted. The water absorption ratio (Q) was determined by following formula

$$Q{\rm{ }} = {\rm{ }}\left( {Wswollen{\rm{ }} - {\rm{ }}Wdry} \right){\rm{ }}/{\rm{ }}Wdry{\rm{ }} \times {\rm{ }}100\%.$$

### Analyzing the rheological properties

Using a 35 mm diameter parallel plate geometry with a 1.0 mm gap, dynamic rheological measurements were conducted on a HAAKETM MARS III Rotational Rheometer at 37 ºC. After a minimum of 24 h post hydrogel fabrication, the hydrogel sample was placed onto the measuring geometry. Applying the silicone oil around the measuring geometry’s perimeter helped to reduce the impact of water evaporation on the rheological data.

The shear viscosity behavior of the PNIPAM-PU/CMC@GEM + NIR hydrogel was analysed using the Carreau-Yasuda model, which effectively describes the relationship between viscosity, shear rate, and temperature for polymer systems.

$$\eta \left( {\gamma \>,\>T} \right) = {\eta _0}\left( T \right){\left( {1 + {{\left( {{{{\eta _0}\left( T \right)\gamma \>} \over {\tau \>}}} \right)}^a}} \right)^{{{n - 1} \over a}}}$$

where $\:\eta$ denotes the shear viscosity (Pa: s), $\:{\eta}_{0}$ is the apparent viscosity at zero shear rate (Pa: s), γ represents the shear rate (S^− 1^), T is the absolute temperature (K), τ corresponds to the temperature to the approximate critical shear stress, A denotes the breadth of the transition zone, and n is the power law exponent.

To account for temperature dependence, the Williams-Landel-Ferry (WLF) model was applied, enabling time-temperature superposition. Model parameters were obtained via inverse fitting.

$$log\left( {{a_T}} \right) = log\left( {{{{\eta _{0\>}}\left( T \right)} \over {{\eta _{0\>}}\left( T \right)}}} \right) = \>{{{C_{1\>}}(T - {T_0})} \over {{C_{2\>}} + (T - {T_0})}}$$

Where T_0_ represents the reference temperature used to develop the compliance master curve, and *C*_*1*_ and *C*_*2*_ are empirical constants adjusted to match the values of the time-temperature superposition factor *a*_t_. Both *C*_*1*_ and *C*_*2*_ are positive and vary depending on the material properties and the selected reference temperature.

where *T₀* represents the reference temperature used to develop the compliance master curve, and *C₁* and *C₂* are empirical constants adjusted to match the values of the time–temperature superposition factor *aₜ*. Both *C₁* and *C₂* are positive and vary depending on the material properties and the selected reference temperature.

### Drug loading capacity determination

To determine the drug loading capacity of GEM in the PNIPAM-PU/CMC@GEM hydrogel, 1 mg of GEM was dissolved in 1 mL of deionized water and incorporated into the hydrogel matrix during the fabrication process. Post hydrogel formation, the unbounded GEM was separated via centrifugation at 15,000 rpm for 15 min at 4 ºC. The supernatant containing free GEM was collected, and its concentration was determined by measuring the absorbance of GEM using a UV-Vis spectrophotometer. The drug loading capacity was calculated using the following formula

$$\eqalign{& L\left( {{{mg\>drug} \over {GEM - PU/CMC\>\>hydrogel}}\>} \right) \cr& = {{{W_{Ed}}} \over {{W_{GEM - PU/CMC\>\>hydrogel}}}} + {{{W_{Td}} - {W_{SD}}} \over {{W_{GEM - PU/CMC\>hydrogel}}}} \cr} $$

In this context, W_Ed_ represents the amount of encapsulated drug, W_TD is_ the total drug content in the hydrogel, W_SD_ refers to the quantity of unencapsulated drug found in the hydrogel.

The loading capacity was analysed to estimate Lmax using the nonlinear curve fitting technique in OriginPro 9.0, based on the Langmuir equation.

$$\:L=\frac{{L}_{\text{max}K\left(D\right)}}{1+K\left(D\right)}$$

Here, D is the concentration of drug (GEM), K is the Langmuir sorption constant, and L_max_ is the maximal loading capacity.

### In vitro drug release study

To evaluate the release profile of GEM from the fabricated PNIPAM-PU/CMC@GEM hydrogel, 1 mg of GEM was dissolved in 1 mL of deionized water and loaded into the PNIPAM-PU/CMC@GEM hydrogel. The hydrogel sample was then placed inside a dialysis bag and immersed in 50 mL of PBS (pH 7.4) maintained at 37 ºC with continuous agitation at 100 rpm. To assess the impact of NIR irradiation on GEM released, the hydrogel was exposed to 808 nm NIR light at 1-hour intervals, using a laser with a power density of 1.5 W/cm^2^. At predefined time points, 1 mL aliquots of the release medium were withdrawn and immediately replaced with 1 mL of fresh PBS to maintain the constant volume and sink conditions. The concentration of released GEM from hydrogel was quantified using UV-Vis spectroscopy by measuring the absorbance at 270 nm. Cumulative release percentage was calculated and plotted to analyze the release kinetics and to assess the effect of NIR exposure on GEM release from the hydrogel matrix.

### In vitro drug release kinetics

To evaluate the release behaviour of GEM from the hydrogel with and without NIR, 5 mg of hydrogel was dispersed in 5 ml of PBS and sealed in dialysis bag (molecular weight cut off: 3500Da). The dialysis setup was maintained at 37 ºC under gentle shaking to mimic physiological conditions. For NIR responsive release evaluation, one set of PNIPAM-PU/CMC@GEM hydrogel was exposed intermittently to NIR irradiation, while other was kept in the dark without NIR. At predetermined time intervals, 5 mL of the external release medium was withdrawn and immediately replenished with an equal volume of fresh PBS to maintain sink conditions. The amount of GEM released was quantified using a UV-vis spectrophotometer and the release profile was compared between NIR-treated and untreated groups.

### Hydrogel concentration, laser power and stability under photothermal conditions

For the purpose of measuring room temperature fluctuations, we made a cylindrical hydrogel and attached a thermometer with a thermocouple to it. After that, it was heated every 10 s while subjected to NIR irradiation at a power of 1.5 W/cm^2^ for 5 min. A blank hydrogel was used as a control to examine the photothermal performance of fabricated hydrogels with different amounts of PNIPAM-PU/CMC@GEM. The hydrogel was subjected to NIR at the wavelength of 808 nm at a power density of 1.5 W/cm^2^ for 5 min, after which it was cooled to its original temperature in order to examine its ability under photothermal conditions. With the blank hydrogel used as a control, thermocouple-equipped thermometer was introduced into the hydrogel and its temperature was recorded every 10 s at room temperature.

### pH and thermoresponsive swelling ratio of PNIPAM-PU/CMC@GEM hydrogel

The swelling behavior of PNIPAM-PU/CMC@GEM hydrogel was studied at various pH levels (1.2, 4.5, 6.5, and 7.4) and temperature (25 to 55 ºC) using buffer solutions. The possible low critical solution (LCST) of the hydrogel sample was the focus of this inquiry. The lyophilized hydrogel was allowed to swell for 48 h by immersion in PBS with the pH 7.4. Subsequently, the inflated samples and humid filter paper was used to wipe off any excess water on hydrogel surface. The swelled hydrogel was removed after a specific period of time, and any remaining fluid on their surface was drained using filter paper. The swelling ratio was calculated by the following formula;

$$\:\text{S}\text{w}\text{e}\text{l}\text{l}\text{i}\text{n}\text{g}\:\text{r}\text{a}\text{t}\text{i}\text{o}\text{n}\:\left(\text{\%}\right)=\frac{{W}_{t}{-W}_{0}}{{W}_{0}}x\:100$$

### In vitro studies

#### Cell viability assay

Using the MTT (3 (4, 5-dimethylthaizol-2-yl)-2, 5-diphenyltetrazoilum bromide) assay, the cytotoxicity of GEM loaded PNIPAM-PU/CMC hydrogel was examined in KYSE-140 cell lines. In brief, 100 µL of fresh DMEM medium and 10 µL of sterile MTT stock solution (5 mg/mL in PBS) were added to the medium after the cells had been incubated with a combination of 100 µL of DMEM and 100 µL of release buffer containing GEM loaded hydrogel at 37 ºC for 24 h. After 4 h of incubation, the unreacted dye was removed via aspiration. The formazan crystals were dissolved by adding DMSO (150 µl/well). Then, using a microplate reader (Infinite M200 PRO, TECAN), the absorbance of the solution was measured at 570 nm. The IC_50_ values were calculated as 52.85 ± 2.5, 90.97 ± 3.6, 60.76 ± 2.8 and 41.59 ± 2.4 µg/mL for GEM, PU/CMC, PNIPAM-PU/CMC@GEM, and PNIPAM-PU/CMC@GEM + NIR respectively.

Similarly, biocompatibility of GEM, PU/CMC, PNIPAM-PU/CMC@GEM and PNIPAM-PU/CMC@GEM + NIR was evaluated on normal esophageal epithelial cell line Het-1 A through MTT assay. Briefly, after treating Het-1 A cells with 100 µL of fresh DMEM medium combined with 100 µL of release buffer containing GEM, PU/CMC, PNIPAM-PU/CMC@GEM and PNIPAM-PU/CMC@GEM + NIR, the cells were incubated at 37 ºC for 24 h. Subsequently, MTT was added and further investigated for the viability as mentioned above.

#### Morphological alterations in the cells after exposed to hydrogel

To evaluate the anti-proliferative and cellular toxicity of GEM, PU/CMC, PNIPAM-PU/CMC@GEM and PNIPAM-PU/CMC@GEM + NIR on KYSE-140 ESCC cancer cell line, AO-EB staining was performed. The cell lines (2 × 10^4^ cells/mL) were cultivated in 96-well plate and exposed to GEM, PU/CMC, PNIPAM-PU/CMC@GEM and PNIPAM-PU/CMC@GEM + NIR. Following a 24-hour incubation period, cells were broken down using trypsin (20 µL/well), washed with PBS, and then stained with 4 µL of AO-EB solution (100 µg/mL). For fluorescence microscopy, the labelled cells were placed on glass slides. When AO enters live cells, it causes green fluorescence, but EB binds to DNA in dead cells, making them glow red. This technique uses the fluorescence colour of cells distinguish between live and dead cells. In 48 well plates, cell lines were grown in DMEM media at a density of 1 × 10^5^ cells/well in order to study nucleus morphological alterations. The cells were exposed to GEM, PU/CMC, PNIPAM-PU/CMC@GEM and PNIPAM-PU/CMC@GEM + NIR and had been incubated for 24 h. The cells were maintained at room temperature for 24 h, and they were stained with DAPI for 10 min at 37 ºC. After washing with PBS to remove excess dye, and they were examined under a fluorescence microscope to look for nuclear changes.

#### Cell migration study by wound scratch test

To evaluate cell migration, a wound closure assay was performed. For the analysis, the KYSE-140 cells were seeded in 6-well plates at a density of 10^6^ cells/well and cultured for 4 days until a confluent monolayer was formed. A linear scratch was then introduced using sterile micropipette tips, followed by gentle rinsing with PBS to remove any detached cells or debris. The cells were subsequently treated with GEM, PU/CMC, PNIPAM-PU/CMC@GEM and, PNIPAM-PU/CMC@GEM + NIR for 24 h. Untreated cells served as negative control and epidermal growth factor (EGF) at 20 µg/mL/10% FBS, which is well known to promote cell migration was used as positive control.

#### Intracellular measurement of ROS production

To investigate the impact of GEM, PU/CMC, PNIPAM-PU/CMC@GEM and PNIPAM-PU/CMC@GEM + NIR on oxidative stress damage, we stained cells 2’,7’-dichloroflurescin diacetate (DCFH-DA). The cells that were treated with GEM, PU/CMC, PNIPAM-PU/CMC@GEM and PNIPAM-PU/CMC@GEM + NIR were washed with PBS and subsequently incubated in serum-free medium containing 10 µM DCFH-DA at 37 ºC for 30 min. Following incubation, trypsin was employed to dissociate the cells, which were subsequently resuspended in PBS for examination. Intracellular ROS generation was observed using a fluorescent microscope and fluorescence intensity was measured to assess ROS levels.

#### Assay for mitochondrial transmembrane potential

To determine the mitochondrial transmembrane potential, the JC-1 fluorescent probe was employed in which healthy mitochondrial cells emit green fluorescence while damaged mitochondria emit red fluorescence. For the analysis, the cells were cultivated in 6 well plates and treated with GEM, PU/CMC, PNIPAM-PU/CMC@GEM and PNIPAM-PU/CMC@GEM + NIR. Following 12 h exposure, cells were stained for 30 min in the culture media with JC-1 (2 µg/mL). Before being viewed under a fluorescent microscope with a UV filter (450–490 nm), adherent cells were rinsed with PBS, detached with trypsin-EDTA, collected in PBS, cleaned by centrifugation, re-immersed in PBS, mixed and finally examined under the microscope.

#### Flowcytometry analysis

The effectiveness of anticancer composites can only be understood by quantifying apoptosis. The process of apoptosis was assessed by means of Annexin V-FITC and PI dual labelling. Cell lines were grown in 6-well plates using 2 mL of DMEM medium, with 2 × 10^5^ cell/well. The incubation medium was changed to 2 mL of new media containing 50 µM GEM, PU/CMC, PNIPAM-PU/CMC@GEM and PNIPAM-PU/CMC@GEM + NIR after 24 h. Control was maintained without any treatment. Annexin V-FITC/PI staining assay kit treated cells were subjected to flow cytometry analysis (Dakewe EXFLOW-206, Shenzhen, China) after an additional 24 h.

### In vivo studies

#### Animal grouping and treatment

Following the acclimatization period, animals were randomly assigned to Groups I, II, III, IV and V. Each group was maintained with 10 animals. Animals in Group I received normal saline as a control or Sham. Group II mice given GEM intratumorally, while group III animals were given PU/CMC. Animals maintained in Group IV and V receives PNIPAM-PU/CMC@GEM and PNIPAM-PU/CMC@GEM + NIR treatment respectively. A volume of 100 mg/mL of GEM, PU/CMC, PNIPAM-PU/CMC@GEM and PNIPAM-PU/CMC@GEM + NIR per mouse was administered once every 3 days, for a total of four injections over the treatment period. In addition, for NIR-treated groups, tumours were irradiated with an 808 nm laser at a power density of 1 W/cm^2^ for 5 min per session, immediately following each injection. The NIR source was placed approximately 1 cm from the tumor surface.

#### In vivo anti tumor therapy

For the purpose of producing xenograft tumor mice, 8 weeks old albino mice with both sexes with the weight of 25 ± 2 g were subcutaneously injected with KYSE-140 cells in the right flank region at a density of 1 × 10^6^ cells. For all animal research, we adhered to the guidelines set out by Animal Ethics Committee of Fujian Medical University, China. Animals were monitored until the tumor size reaches 150 mm^3^. Animals were divided into five groups as mentioned above. Experiment was carried out for 30 days, and each formulation were intravenously injected for every three days. The caliper was used to measure the tumor size weekly twice. Following this, the tumor volume was calculated using the following formula,

$$V{\rm{ }} = {\rm{ }}\left( {length} \right){\rm{ }} \times {\rm{ }}{\left( {width} \right)^2}/2$$

At the end of the experiment, the tumors were surgically removed from the subcutaneous armpit and the mice were euthanized via CO_2_ inhalation.

#### In vivo toxicity analysis

Blood samples were extracted from the treated animals and stored at 36 ºC for 2 hours. After that, the blood sample was centrifuged at 1000 rpm for 15 minutes to separate the serum. Subsequently, the blood samples were analysed for the following blood biochemical parameters such as SGOT, SGPT, ALP, urea and creatinine using biochemical assay kit as per the manufacturer’ instructions.

#### qRT-PCR analysis

Notable genes such as MAPK, VEGFA, BCL-2, MAPK, HIF-1α and PDGF were chosen to validate the mRNA expression level after treated the tumor cells with GEM, PU/CMC, PNIPAM-PU/CMC@GEM and PNIPAM-PU/CMC@GEM + NIR. qRT-PCR was used to measure the expression of mRNA level after eight weeks following injection. The tumor cells acquired from mice were homogenized and mixed with bioZol reagent before RNA extraction. Using a cDNA filter column, the DNA was extracted from the cell solution. After the filtrate was transferred to an RNA pure column, it was centrifuged at 12, 000 rpm for 30 s. The RNA was then mixed with specific reverse primers obtained from Vazym, Nanjung, China. For the analysis, the Quantstudio 1 RT-PCR equipment (Applied Biosystems, Thermoscientific, USA) was used, and the one-step qRT-PCR kit was used in accordance with the manufacturer’s instructions. For this study, we used a SYBER Green Master Mix kit along with appropriate primers.

#### Histopathology analysis

The tumor tissues, along with the kidney, heart, liver, spleen, and lungs were removed and subsequently preserved in 10% neutral buffered formalin for overnight to maintain tissue architecture. The fixed samples were treated for paraffin embedding, sectioned into 5 μm-thick longitudinal slices with a microtome, and then deparaffinized using xylene and rehydrated through ethanol. For histological evaluation, the slices were stained with Hematoxylin solution for 3 min, subsequently differentiated in HCL-ethanol (1:7) for 10 s and thoroughly rinsed with distilled water. The slides were subsequently counterstained with eosin solution for 10 min, dehydrated in ethanol, cleaned with xylene and mounted with neutral balsam. The stained sections were analysed using an inverted microscope to assess histopathological alterations, cellular morphology, and tissue integrity.

### Statistical analysis

Statistical analysis was conducted using Prism 5 (GraphPad, San Diego, CA). All experiments were performed in triplicate, and data are presented as mean ± SD. Statistical significance was determined using one-way ANOVA followed by post-hoc analysis (*p* < 0.05).

## Results

### Hydrogel fabrication and characterization

**Fig. 2:**
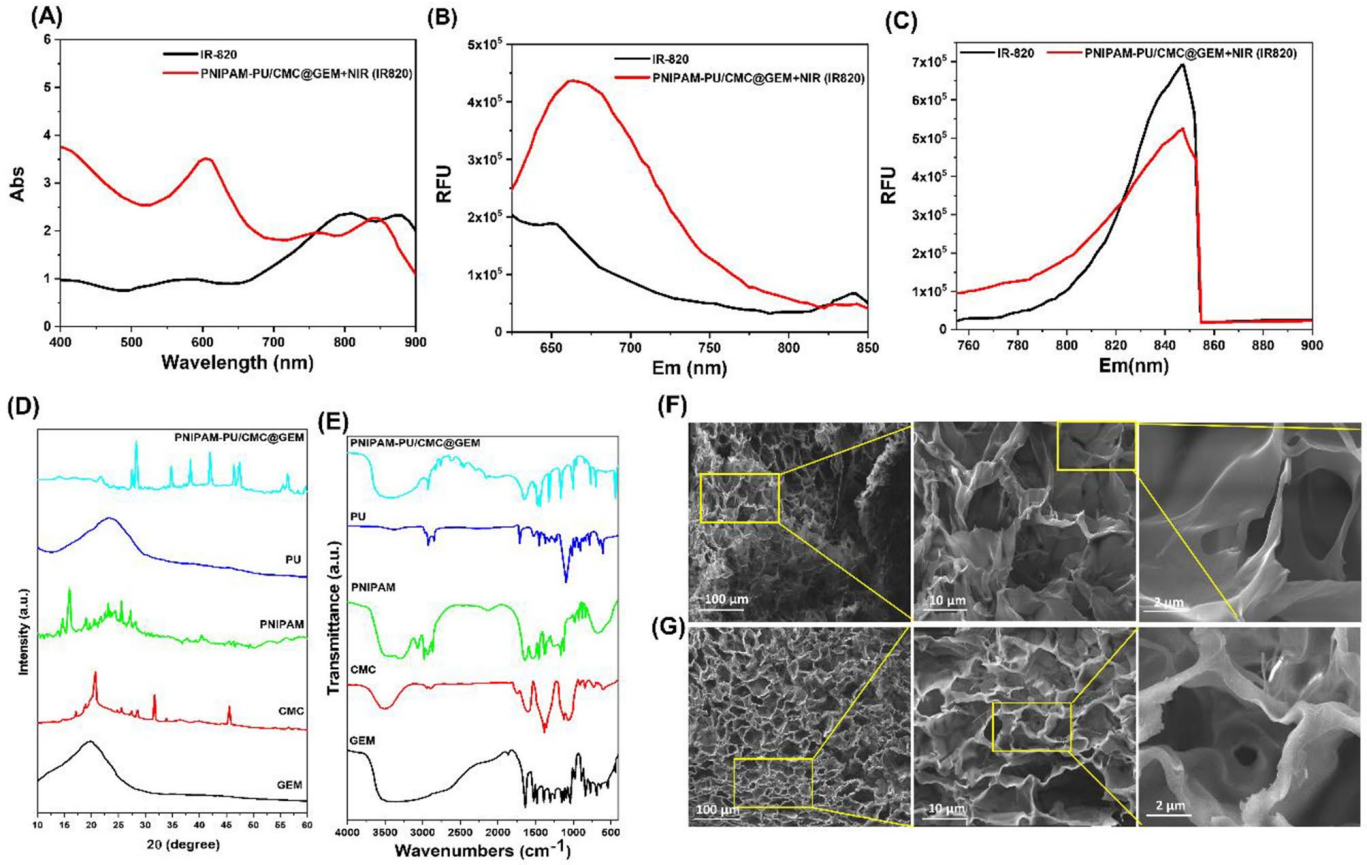
**(A)** UV-Vis spectra analysis of IR-820 and PNIPAM-PU/CMC@GEM + NIR (IR-820); **(B)** Fluorescence emission spectra of IR-820 and PNIPAM-PU/CMC@GEM + NIR (IR-820); **(C)** Fluorescence emission spectra of IR-820 and PNIPAM-PU/CMC@GEM + NIR (IR-820) with an excitation wavelength of 735 nm; **(D)** X-Ray diffraction analysis of GEM, CMC, PNIPAM, PU, PNIPAM-PU/CMC@GEM; **(E)** FT-IR spectra of GEM, CMC, PNIPAM, PU, PNIPAM-PU/CMC@GEM; **(F)** SEM images of PU/CMC and **(G)** PNIPAM-PU/CMC@GEM hydrogel

To enhance the photothermal responsiveness of the hydrogel system, IR-820 dye was incorporated into the PNIPAM-PU/CMC@GEM + NIR hydrogel. As shown in Fig. 1 A, the UV-vis absorption spectrum of the IR-820 infused PNIPAM-PU/CMC@GEM hydrogel displayed two prominent absorption peaks at approximately 580 nm and 850 nm, corresponding to the characteristic absorbance of GEM and the IR-820 dye, respectively. This reveals the successful integration of both the chromotherapeutic agent and the photothermal sensitizer within the hydrogel matrix. In addition, the fluorescence spectrum of the composite hydrogel (Fig. 2 A) showed a distinct emission peak at approximately 670 nm upon excitation at 595 nm, attributed to GEM. Furthermore, a separate emission peak at 840 nm was observed in Fig. 2B & C when excited at 735 nm, which is characteristic of IR-820. These results demonstrate that both therapeutic and photo responsive components retained their optical signatures post-fabrication. More importantly, the fluorescence emission of each component could be selectively detected by tuning the excitation wavelength confirming that the fabricated hydrogel allows for independent optical monitoring of both GEM and IR-820. This dual functionality supports the potential of hydrogel for combined chemo-photothermal therapy in cancer treatment.

X-ray diffraction analysis was performed to determine the crystalline nature and structural composition of PU, CMC, PNIPAM, GEM and PNIPAM-PU/CMC@GEM hydrogel. As depicted in Fig. 2D, the CMC and PNIPAM displayed characteristic semi-crystalline features with peaks at 20.3º and 22.5º respectively, which are consistent with their partially ordered molecular structures. PU displays a broad halo centered around 18º-22º, indicative of an amorphous polymeric phase. In the PNIPAM-PU/CMC@GEM hydrogel, the prominent crystalline peaks of GEM were significantly reduced in intensity, particularly at 23.6º and 27.8º, suggesting that successful encapsulation of GEM within the hydrogel matrix. These results authenticate the successful integration of GEM into the PNIPAM-PU/CMC hydrogel while maintaining the structural integrity of the hydrogel.

The FTIR spectra of PU, PNIPAM, GEM, CMC and PNIPAM-PU/CMC@GEM were analysed to confirm the successful fabrication of components in the hydrogel. As shown in Fig. 2E PU displays sharp peak at approximately 1721 cm-1 and 1530 cm-1, reveals the urethane carbonyl carbon (C = O) and NH stretching of urethane respectively [31]. The absorbance peak at 1628 cm-1 corresponds to the existence H bonded carbonyl carbon (C = O) [38], which is found in both prepolymer and copolymer. FTIR spectra of PU/CMC are displayed in Fig. 2E. The expression of peaks around 3320 cm-1 corresponds to the stretching vibration of OH and NH groups. Further, the bands at 2960, 1710, and 1261 cm-1 were responsible to the bending vibration of carboxylic groups in CMC at 1599 and 1420 cm-1 respectively. The emergence of peaks at around 1599 and 1420 cm-1 corresponds to the stretching vibration of asymmetric modes of carboxylic groups. The stretching vibration of NH group was overlapped with symmetric stretching vibration of carboxylic groups in CMC.

The morphology of the fabricated hydrogel was evaluated using FESEM analysis. The hydrogel exhibited a consistent porous structure. The PNIPAM-PU/CMC@GEM hydrogel exhibited well connected pores with consistent morphology in comparison to the PU/CMC hydrogel (Fig. 2 F & G). The pore diameter was measured as 46.96 ± 12.94 μm for PU/CMC hydrogel and 42.96 ± 13.49 μm for PNIPAM-PU/CMC@GEM hydrogel reveals that, the drug loaded PNIPAM-PU/CMC hydrogel displays high porous structure. Furthermore, the PNIAPM-PU/CMC@GEM hydrogel demonstrated a more compact consistency due to its superior water retention capacity, significantly improves the controlled and prolonged release of drugs through a remarkable swelling transition. All materials displayed sheet or sponge-like configurations. Furthermore, PNIPAM-PU/CMC@GEM hydrogel possess multiple interconnected pores, facilitating the natural shrinkage process to extract a substantial volume of water.

### Rheological studies

**Fig. 3:**
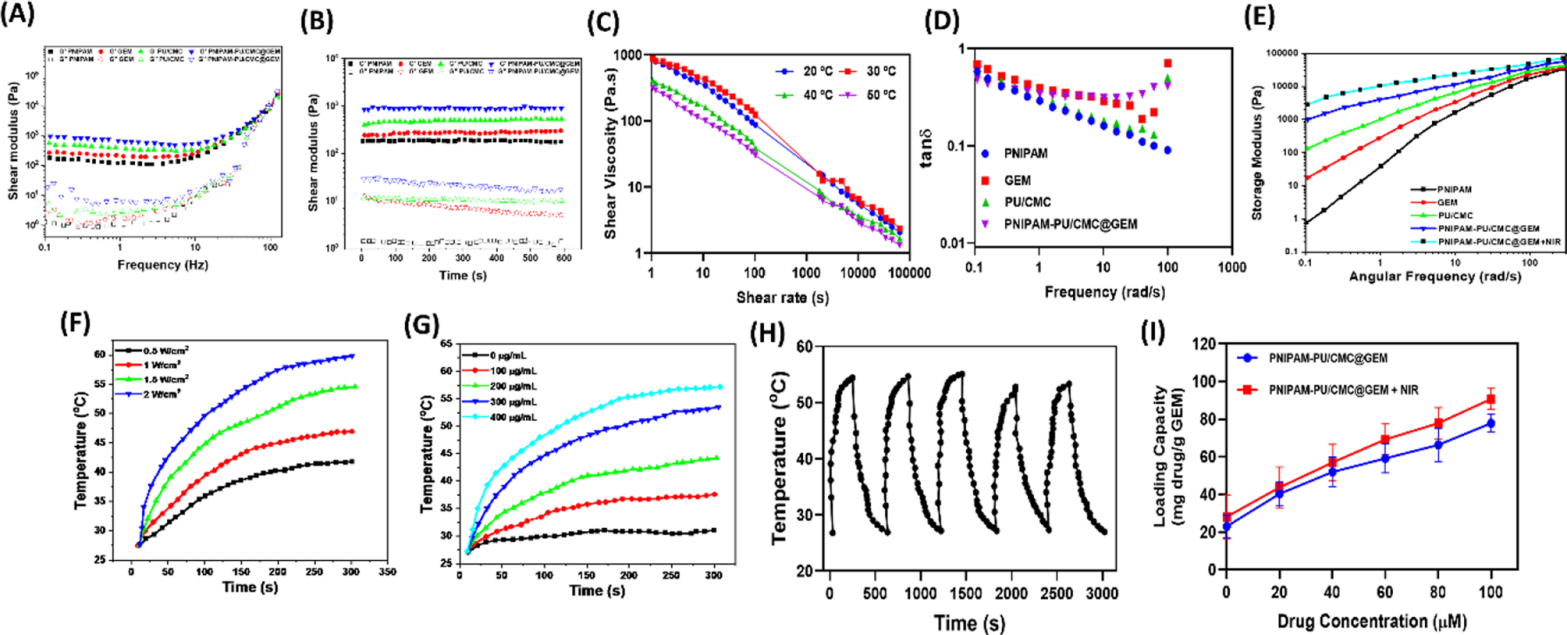
**(A)** Frequency dependent analysis of the fabricated hydrogels ranging from 0.1 to 100 Hz; **(B)** Variations in the moduli of the hydrogel at different time; **(C)** Shear viscosity of experimental results and Carreau-Yasuda model fitting curves at different temperatures; **(D)** Frequency-dependant variation of the loss tangent (tan 𝛿) for different hydrogel formulations; **(E)** Effect of angular frequency on the storage and loss moduli of fabricated hydrogel; **(F)** Hydrogels infused with varying concentrations of PNIPAM-PU/CMC@GEM were exposed to NIR laser irradiation at a power density of 1.5 W/cm² to observe their response; **(G)** The temperature of the hydrogels was monitored over time under irradiation with an 808 nm laser, demonstrating their photothermal performance; **(H)** A temperature profile was generated for hydrogels containing PNIPAM-PU/CMC, highlighting their heat generation capacity under 1.5 W/cm² NIR laser exposure and **(I)** Drug loading capacity of GEM on fabricated hydrogel with and without NIR

The rheological evaluation of PU/CMC demonstrates distinct microstructural behaviour relative to pure PU as a function of angular frequency, influenced by the interaction between PU and CMC. As shown in in Fig. 3A & B, the rheological testes revealed that the hydrogel has shear thinning capabilities. The storage modulus, G’ was greater that the loss modulus, while G’’, proving that the gel; was stable and would maintain its integrity upon injection. Important for preserving therapeutic qualities, physicochemical characterization verified the effective production and incorporation of PNIPAM-PU/CMC@GEM into the hydrogel matrix. Due to its shear-thinning characteristic, hydrogels are ideal for injectable applications because they facilitate both the ease of administration and the generation of gels in situ. The frequency dependant storage modulus (G’), loss modulus (G”), and complex viscosity were determined at 37 ºC in dynamic mode and illustrated in Fig. 3C. All solutions demonstrate classic gel like properties, with PNIPAM-PU/CMC@GEM exhibiting a greater comparable gradual decrease in viscosity, which aligns with the expectation that viscosity consistently diminishes as frequency increases. Further, the rheological analysis was conducted by fitting the shear viscosity as a function of shear rate and temperature using Carreau-Yasuda and WLF models. The model parameters were obtained through an inverse fitting approach. As shown in Fig. 3C, the Carreau-Yasuda model provided an excellent fit across the tested shear rate and temperature ranges. The experimental data exhibited strong agreement with the model predictions under all evaluated conditions, confirming the reliability of the rheological characterization. The viscosity behaviour is almost linear, with a slight decrease in viscosity over an extended time scale for both PU/CMC and PNIPAM-PU/CMC@GEM, attributed to the loosely ordered structure resulting from the shearing effect. The integration of PNIAPM in PU significantly enhances the shear viscosity of PNIPAM-PU/CMC relative to PU/CMC hydrogel. Additionally, PNIPAM-PU/CMC@GEM + NIR hydrogel displays a frequency dependant viscoelastic behaviour. Specifically, the storage modulus (G’) increased with rising frequency, while the loss modulus (G’’) remained relatively stable or slightly decreased. This was further supported by reduction in the damping factor (tan δ G’’/G’) across the tested frequency range (Fig. 3D), which decreased by a factor of 6.7, 4.9, 3.4 and 1.2 for GEM, PU/CMC, PNIPAM-PU/CMC@GEM and PNIPAM-PU/CMC@GEM + NIR respectively. The frequency dependant viscosity is displayed in Fig. 3E. These findings reveals that the fabricated hydrogel exhibits pronounced solid-like behaviour at higher frequencies, while at lower frequencies, the hydrogel more like a viscoelastic fluid. This behaviour implies that at lower frequencies, there is sufficient time for network rearrangements and relaxation of dynamic interactions, leading to increased flowability. In contrast, at higher frequencies, the polymeric network has less time to recognize, thereby maintaining its structure and demonstrating a stiffer, more solid like response. Owing to their superior hydrophilic-hydrophobic balance and well-defend molecular structure, the fabricated hydrogel can be considered as enhanced drug delivery vehicle.

### Photothermal characteristics

Upon exposure to 808 nm laser, the temperature of the hydrogel system rapidly elevated within 5 min, with the magnitude of the increase directly correlated to the concentration. Higher concentrations resulted in more noticeable temperature increase, according to the time and temperature increases. As shown Fig. 3G, after 5 min the temperature reached 54.4 ºC for a concentration of 300 µg/mL. The photothermal response of fabricated PNIPAM-PU/CMC@GEM hydrogel was examined using a range of power settings. The hydrogel’s temperature was increased in response to a rise in power. The temperature increased from 31.96 ºC to 54.71 ºC at a power of 1.5 W/cm^3^ for 5 min, the PNIPAM-PU/CMC@GEM hydrogel was left to cool down to its original temperature naturally (Fig. 3F). In order to monitor the temperature variations over time, this process was repeated for 5 times. The findings displayed that, the hydrogel kept their photothermal stability even after several times of heating and cooling cycles, with no noticeable decrease in photothermal conversion efficiency (Fig. 3H). The integration of thermoresponsive polymers, such as PNIPAM, with NIR light offers a dual benefit; the temperature induced phase transition of the PNIPAM-PU/CMC@GEM hydrogel can be precisely adjusted to regulate drug release, while NIR irradiation serves as non-invasive, remote mechanism to locally elevate the temperature and facilitate drug release at the targeted location. This technique offers substantial potential for realizing targeted therapy with less adverse effects. As the drug can be selectively administered to the tumor location upon NIR irradiation, thereby stimulating systemic dispersion (Fig. 3H). Further, the capacity to precisely regulate the release of GEM in reaction to external NIR exposure indicates that this approach may be further refined for clinical use, especially in treating tumors that necessitate targeted drug delivery with reduced systemic toxicity.

### In vitro drug loading, release profile and kinetics

Furthermore, the drug loading capacity of both PNIPAM-PU/CMC@GEM and PNIPAM-PU/CMC@GEM + NIR formulations exhibited a concentration-dependent increase, likely due to the progressive saturation of available encapsulation sites. Notably, NIR treatment significantly enhanced the drug loading efficiency of the hydrogel, which can be attributed to photothermal-induced matrix softening and increased polymer chain mobility, facilitating deeper GEM entrapment (Fig. 3I). The maximum loading capacities of both formulations were determined by fitting the experimental data to a Langmuir isotherm model, and the results are presented in Table 1.

**Table 1:** Association constants, static quenching constants, loading capacity and entrapment efficiency of GEM in PNIPAM-PU/CMC@GEM and PNIPAM-PU/CMC@GEM + NIR

Formulation	K_sv_ ± SE (10^4^ M^− 1^)	V ± SE (10^4^ M^− 1^)	L_max_ ± SE (mg/drug PT-SLNPs)	EE (%) ± SE
PNIPAM-PU/CMC@GEM	1.38 ± 0.08^a^	0.50 ± 0.03^a^	156 ± 14^a^	76.2 ± 2.5
PNIPAM-PU/CMC@GEM + NIR	1.58 ± 0.16^a^	0.56 ± 0.03^b^	178 ± 12^b^	83.4 ± 3.4

**Fig. 4:**
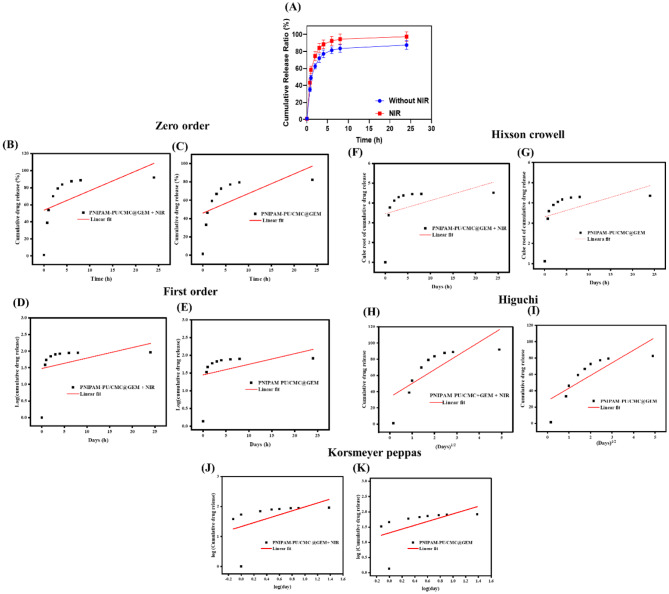
(**A**) In vitro drug release profile; (**B**-**K**) Drug release kinetics: Release kinetics simulations of PNIPAM-PU/CMC@GEM with NIR and without NIR with zero order, first order, Higuchi, and Korsmeyer Peppas

The release of GEM from the hydrogel followed a diffusion-controlled pattern, with 25% released over 24 h at 37 ºC, as demonstrated in Fig. 4 A, when NIR irradiation was not present. Cumulative release increased to 75% over 24 h under NIR exposure (808 nm, 1-hour intervals, 1.5 W/cm^2^), denotes the effective drug release after NIR therapy. To measure the concentration of GEM and to chart its release profile overtime, absorbance values were utilized. The hydrogel that was exposed NIR light had a steeper slope than the control group, suggesting that the release of GEM was accelerated and controlled by the light. The rapid release can be attributed to the hydrogel’s thermoresponsive PNIPAM, which cause localized heating. Rapid drug release is achieved when the hydrogel matrix is either expanded or its connections with GEM were disrupted by the heat generated by PNIPAM [39]. Thermoresponsive hydrogel improves passive diffusion, which in turn create micro cavities in the hydrogel, which release GEM even more efficiently [40]. The use of NIR light as a remote trigger for regulated medication release for enables modification according to therapeutic requirements [41]. This method helps to release large amount of GEM at the tumor site while avoiding systemic toxicity makes it a promising tool for cancer therapy. A larger regulated dose of GEM delivered by enhanced release under NIR irradiation, can have a greater therapeutic impact against tumor cells with fewer side effects.

The ability to regulate drug release via external NIR stimuli presents a promising approach for localized cancer therapy, allowing for a higher concentration of GEM to be delivered at the tumor site while minimizing systemic toxicity [42]. This controlled release mechanism not only improves drug bioavailability but also reduces off-target effects, thereby enhancing therapeutic efficacy. The integration of NIR-responsive hydrogels for GEM delivery represents a potential advancement in targeted chemotherapy, offering a non-invasive and adjustable treatment strategy for improved clinical outcomes. Therapeutic efficacy is enhanced by reducing non-targeted effects and improving medication absorption through this controlled release method [43]. Under NIR stimulation, the in vitro drug release from the PNIPAM-PU/MC@GEM hydrogel followed the Korsmeyer-Peppas model with a strong fit (R^2^ = 0.94), indicating controlled release (Fig. 4B-K). The release exponent (*n* = 0.679) revealed an anomalous transport mechanism involving both diffusion and polymer relaxation. NIR exposure enhanced this process, likely due to localized heating that altered the hydrogel’s structure, promoting faster drug diffusion. A higher release rate constant (K = 0.53) under NIR further supports the role photothermal effects in accelerating release (Table 2). Overall, the Korsmeyer–Peppas model effectively captures the enhanced and modulated drug release behavior under NIR irradiation, validating the potential of NIR-responsive hydrogels as smart drug delivery systems [44]. These findings confirms that the PNIPAM-PU/CMC@GEM + NIR hydrogel’s-controlled drug delivery is ideal for therapeutic applications. Hence, for potential therapeutic outcomes, the incorporation NIR and thermoresponsive hydrogels with GEM administration offers a non-invasive and adaptable treatment approach for targeted drug delivery.

**Table 2:** Drug release kinetics and R^2^ values of PNIPAM-PU/CMC@GEM + NIR and PNIPAM-PU/CMC@GEM hydrogel

Kinetics model	PNIPAM-PU/CMC@GEM + NIR		PNIPAM-PU/CMC@GEM	
	*R* ^2^	K (constant)	*R* ^2^	K (constant)
Zero order model	0.81	5.99	0.74	0.531
First order model	0.86	0.59	0.813	0.38
Hixson crowell	0.64	0.066	0.59	0.061
Higuchi model	0.96	0.281	0.91	0.253
Korsmeyer peppas model	0.94	0.532	0.91	0.457

### In vitro cytotoxicity assay

**Fig. 5:**
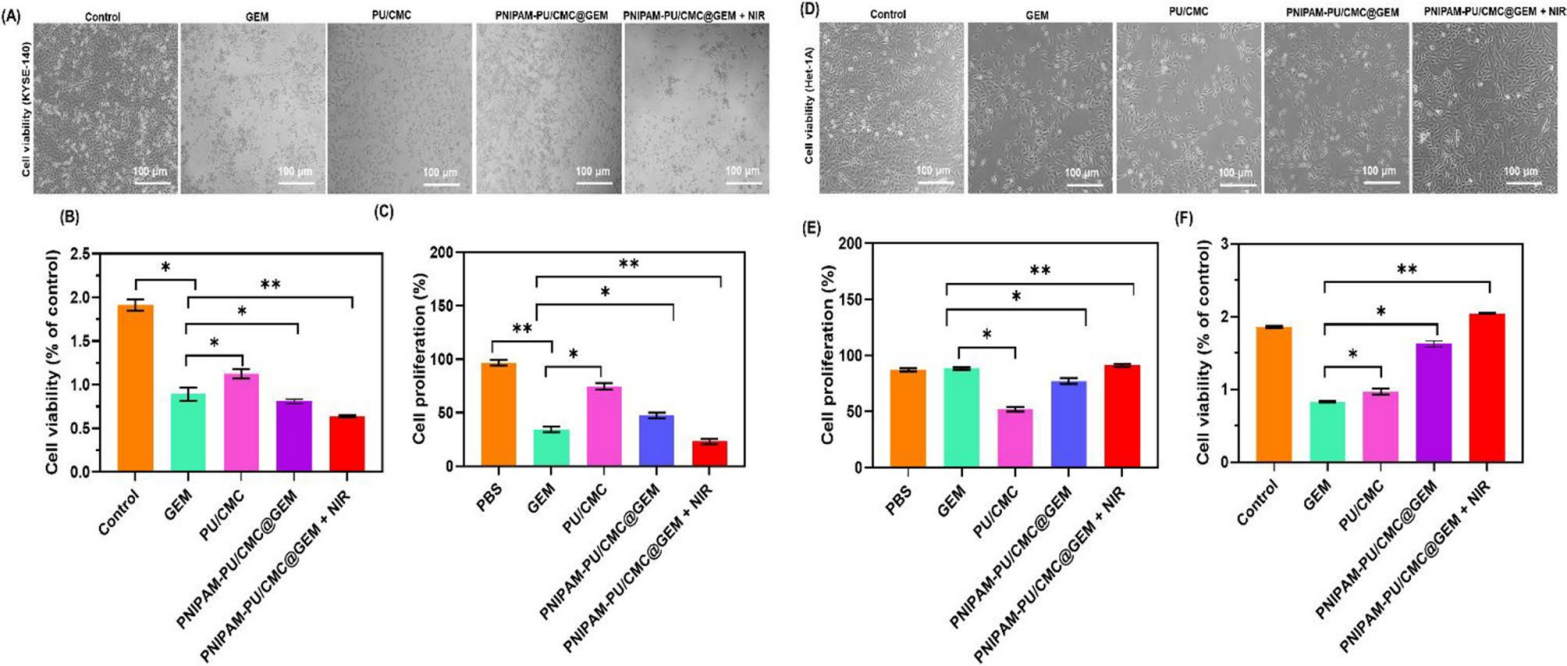
**(A)** Cell viability of KYSE-140 cells after exposed to GEM, PU/CMC, PNIPAM-PU/CMC@GEM and PNIPAM-PU/CMC@GEM + NIR; **(B)** MTT assay to quantitatively evaluate the cell viability KYSE-140 after treated with GEM, PU/CMC, PNIPAM-PU/CMC@GEM and PNIPAM-PU/CMC@GEM + NIR; **(C)** Cell proliferation of KYSE-140 cells after treated with hydrogel; **(D)** Cell viability of Het-1 A cells after exposed to GEM, PU/CMC, PNIPAM-PU/CMC@GEM and PNIPAM-PU/CMC@GEM + NIR; **(B)** MTT assay to quantitatively evaluate the cell viability Het-1 A after treated with GEM, PU/CMC, PNIPAM-PU/CMC@GEM and PNIPAM-PU/CMC@GEM + NIR; **(C)** Cell proliferation of Het-1 A cells after treated with hydrogel; Data were presented as mean ± SD (*n* = 3, *p < 0.05)

The cytotoxicity of GEM, PU/CMC, PNIPAM-PU/CMC@GEM, and PNIPAM-PU/CMC@GEM + NIR was investigated on KYSE-140 cells using the MTT test, with the help of Transwell inserts technology. The IC_50_ values were calculated as 52.85 ± 2.5, 90.97 ± 3.6, 60.76 ± 2.8 and 41.59 ± 2.4 µg/mL for GEM, PU/CMC, PNIPAM-PU/CMC@GEM, and PNIPAM-PU/CMC@GEM + NIR respectively (Supplementary Fig. 1). Moreover, the role of photothermal effect of PNIPAM-PU/CMC@GEM following NIR irradiation on KYSE-140 cells was evaluated. After exposure the cells to fabricated hydrogel and GEM, the viability was reduced. However, the NIR irradiation significantly reduce the cell growth and shows enhanced cytotoxic effects compared to others. As shown in Fig. 5 A, when exposed to NIR induced hydrogel, KYSE-140 cells show significant morphological changes and lower viability compared to others. The absorbance at 570 nm showed a considerable decrease in KYSE-140 cells, indicating an increase in anticancer activity during NIR exposure (Figure. 5B). Further, the cell proliferation was significantly reduced when exposed to PNIPAM-PU/CMC@GEM + NIR compared to others (Fig. 5 C). It has been well documented that, NIR cand induce lethal effects by increasing cell sensitivity through hyperthermia mediated apoptosis in cancer cells [45].

In addition, the biocompatibility of the fabricated hydrogel was evaluated on normal human esophageal epithelial cells (Het-1 A) through MTT assay. The results displayed that, minimal cytotoxic effects on Het-1 A cells in all treatment groups (Fig. 5D). Cell viability was observed above 85% in PNIPAM-PU/CMC@GEM treated group. However, NIR irradiation treated group shows 90% viable cells after 24 h exposure (Figure. 5E). The cell proliferation was also elevated in PNIPAM-PU/CMC@GEM + NIR treated group when compared to others (Fig. 5 F). In contrast, GEM treatment alone showed a moderate reduction in viability, suggesting that the hydrogel with NIR effectively reduces the toxicity on non-targeted cells.

**Fig. 6:**
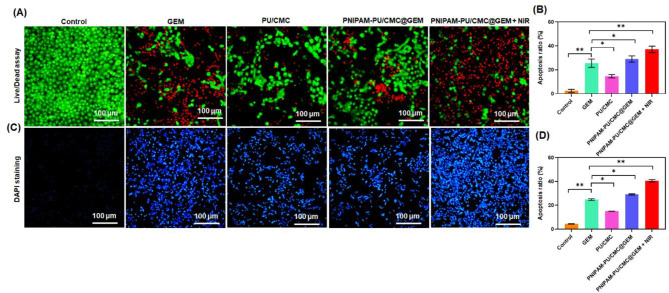
**(A)** Effect of GEM, PU/CMC, PNIPAM-PU/CMC@GEM and PNIPAM-PU/CMC@GEM + NIR on viability of KYSE-140 cells was evaluated through Live-dead cell assay by AO-EB staining; **(B)** Quantitative analysis of dead cells after exposed to GEM, PU/CMC, PNIPAM-PU/CMC@GEM and PNIPAM-PU/CMC@GEM + NIR; **(C)** Nuclear staining of (DAPI) of KYSE-140 cells after treated with GEM, PU/CMC, PNIPAM-PU/CMC@GEM and PNIPAM-PU/CMC@GEM + NIR; **(D)** Quantitative analysis of apoptosis ratio. Data were presented as mean ± SD (*n* = 3, *p < 0.05)

The fluorescent live and dead cells after treated with GEM, PU/CMC, PNIPAM-PU/CMC@GEM and PNIPAM-PU/CMC@GEM + NIR was shown in Fig. 6 A. The quantification of the apoptosis ratio after exposed to hydrogel, NIR therapy and GEM displayed in figure. 6B. The control group cells emit diminished red fluorescence and increased green fluorescence clearly demonstrates the viability of cells in untreated group. The cells exposed to GEM, PU/CMC, PNIPAM-PU/CMC@GEM, and PNIPAM-PU/CMC@GEM + NIR emits increased red florescence and decreased green fluorescence which clearly indicated the cell death. However, NIR induced hydrogel treated cancer cells shows maximum red fluorescence suggesting increased cell death as a result of the cytotoxic effects of PNIPAM-PU/CMC@GEM + NIR which induce ROS production and harm cell membranes.

Figure 6 C, shows the microscopic images of DAPI staining after treated with GEM, PU/CMC, PNIPAM-PU/CMC@GEM, PNIPAM-PU/CMC@GEM + NIR. The KYSE-140 cells in the control group that not receive any treatment had normal nuclei, which were uniformly stained with DAPI and did not show any evidence of nuclear fragmentation or chromatin condensation. In GEM, PU/CMC and PNIPAM-PU/CMC@GEM treated group minor nuclear alterations was observed, while severe nuclear changes, such as enhanced chromatin condensation, nuclear shrinkage, and death, were seen in KYSE-140 cells treated with PNIPAM-PU/CMC@GEM + NIR which displays the cytotoxic potential of NIR irradiated thermoresponsive hydrogel (Fig. 6D). Hence, in the prescence of NIR, the GEM may triggers DNA intercalation and ROS mediated damage when combined with PNIPAM-PU/CMC which increases the apoptosis rate in cancer cells. The nuclear changes were amplified by the NIR treatment, which promoted cell death by apoptosis or necrosis; this was especially true in the PNIPAM-PU/CMC@GEM + NIR group as compared to without NIR group [46]. Thus, fabricated hydrogel caused changes to the nucleus as shown by DAPI staining. The nuclear alterations described here are in agreement with the mechanisms of GEM and PU/CMC respectively, which causes oxidative DNA damage and intercalate into DNA to cause double stand breaks.

The results demonstrate that PNIPAM-PU/CMC@GEM hydrogel exhibits enhanced cytotoxicity against KYSE-140 cells, particularly under NIR irradiation. The significant reduction in cell viability and proliferation upon NIR exposure confirms the synergistic effect of hyperthermia and chemotherapy. The photothermal effect of PNIPAM contributes to increased cellular sensitivity, likely promoting apoptosis through heat-induced mechanisms. Further, NIR induced PNIPAM-PU/CMC@GEM hydrogel displays increased cell death which was evidenced by Live/Dead staining. In addition, DAPI staining displays the increased cell death, nuclear fragmentation and chromatin condensation particularly in the NIR treated group [47]. These effects highlight the synergistic action of GEM-induced DNA intercalation and PU/CMC-mediated oxidative stress, enhanced by NIR induced thermoresponsive hydrogel behaviour [48]. These findings suggest the promising potential of the NIR-responsive hydrogel system for targeted and efficient esophageal cancer therapy.

### Assay for mitochondrial membrane potential analysis

**Fig. 7:**
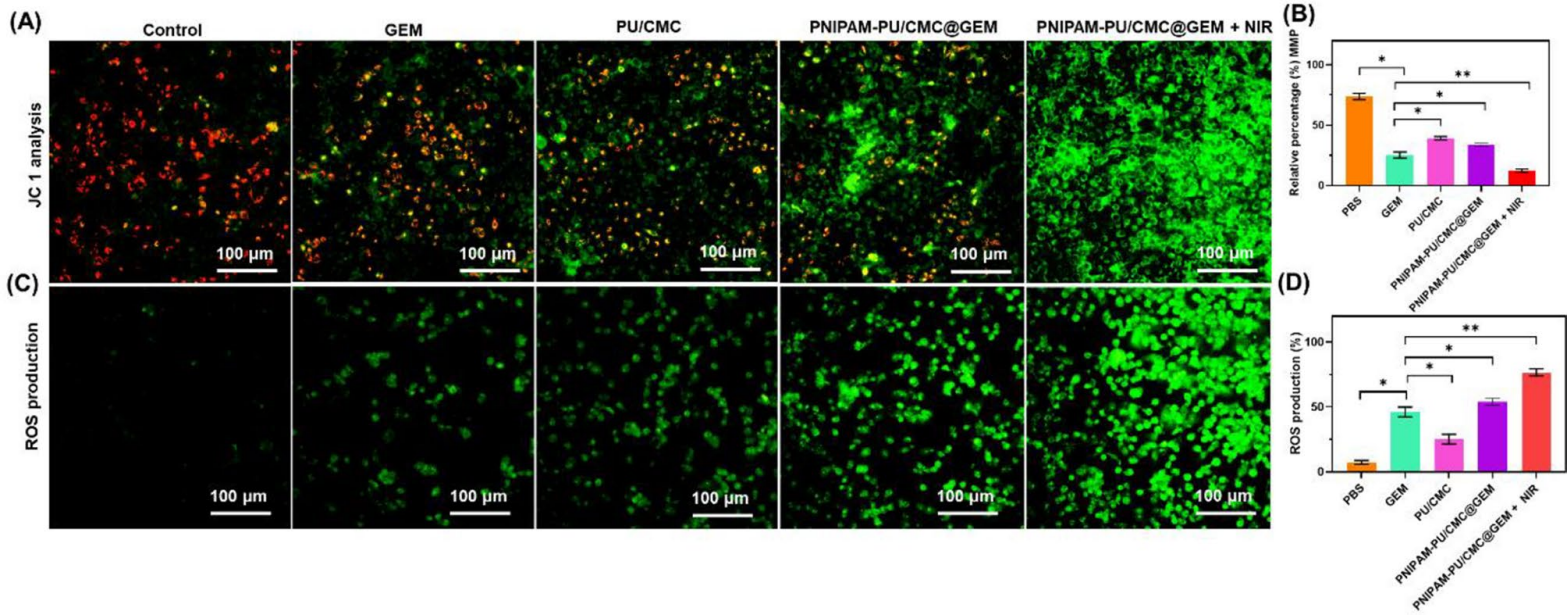
**(A & B)** Mitochondrial membrane potential determined by JC-1 staining. Representative images show MMP in control and GEM, PU/CMC and PNIPAMPU/CMC@GEM with NIR and without NIR. Treated cells show a fluorescence shift from red to green, indicating mitochondrial depolarization. The increased green fluorescence intensity reflects cell damage following exposure to NIR-induced PNIPAM-PU/CMC@GEM; (C & D) Qualitative and quantitative measurement of ROS generation after exposed to GEM, PU/CMC and PNIPAMPU/CMC@GEM with NIR and without NIR was determined by DCFH-DA method. Data were presented as mean ± SD (*n* = 3, *p < 0.05)

MMP is a key factor for mitochondrial function, and its disruption is closely linked to apoptosis in cancer cells. In the present study, the changes in MMP after treated with GEM, PU/CMC, PNIPAM-PU/CMC@GEM, PNIPAM-PU/CMC@GEM + NIR was evaluated by JC-1 analysis to determine the mitochondrial depolarization. The control or untreated cells exhibited strong red orange fluorescence, indicating that the mitochondria were healthy and unharmed. After 24 h of treatment with GEM, PU/CMC, PNIPAM-PU/CMC@GEM, PNIPAM-PU/CMC@GEM + NIR, there was steady drop in red fluorescence and an increase in green fluorescence, indicating partial polarization. A more pronounced red-to-green shift was pronounced red-to-green shift was observed in cells exposed to PNIPAM-PU/CMC@GEM + NIR group, indicating a substantial disruption of mitochondria within 24 h. Figure 7 A & B, show that, the cells treated with PNIPAM-PU/CMC@GEM + NIR showed the highest depolarization and a significant decrease in mitochondrial transmembrane potential (ΔΨm). In cells that were either treated or left untreated, the integrity of ΔΨm was maintained. Increased mitochondrial depolarization and dysfunction were seen in PNIPAM-PU/CMC@GEM + NIR treatments compared to GEM, PU/CMC, and PNIPAM-PU/CMC@GEM, demonstrating that activating NIR enhances apoptotic potential of fabricated hydrogel. The significant mitochondrial disruption can be attributed to the synergistic effect of the hydrogel components (PU, CMC, PNIAPAM) and NIR irradiation. Therefore, it is likely that, the oxidative stress and ROS that caused mitochondrial disruption were amplified by the addition of PU, CMC and GEM. Cancer treatment relies on apoptotic-inducing cumulative mitochondrial damage, as shown by a time dependant increase in depolarization. Additionally, through oxidative stress-induced cancer cell death, both PU/CMC and GEM elevates ROS production. Therefore, PNIPAM-PU/CMC@GEM + NIR appears to be the most promising drug for inducing mitochondrial dysfunction in cancer cells.

The significant mitochondrial disruption observed in the PNIPAM-PU/CMC@GEM + NIR-treated cells can be attributed to the synergistic effects of the hydrogel components and NIR irradiation [49]. The integration of PU and CMC likely facilitated enhanced cellular uptake and retention of GEM, while PNIPAM contributed to controlled drug release in response to external stimuli [50]. Additionally, NIR irradiation has been well-documented to exacerbate ROS generation, leading to oxidative stress-induced apoptosis in cancer cells [51]. The enhanced ROS production observed in the PNIPAM-PU/CMC@GEM + NIR-treated cells is in agreement with previous studies showing that NIR exposure amplifies oxidative stress, further compromising mitochondrial integrity and accelerating apoptotic pathways [52].

### Intracellular measurement of ROS production

ROS levels were quantified in KYSE-140 cells following treatment with GEM, PU/CMC, PNIPAM-PU/CMC@GEM, and PNIPAM-PU/CMC@GEM + NIR through DCFH-DA staining. Figure 7 C represents ROS fluorescence images for untreated cells and GEM, PU/CMC, PNIPAM-PU/CMC@GEM and PNIPAM-PU/CMC@GEM + NIR treated cells. The quantification of ROS generation of KYSE-140 cells was shown in Fig. 7D. The findings displayed that untreated or control cells exhibited negligible amounts of ROS production. The PNIPAM-PU/CMC@GEM + NIR treatment led to a substantial increase in ROS release due to the action of NIR. Furthermore, PNIPAM-PU/CMC@GEM + NIR treated group exhibited the greatest levels of ROS production due to the synergistic effect of GEM, PNIPAM, PU/CMC under NIR exposure. The quantification of ROS demonstrated that, NIR activation markedly elevated ROS generation in PNIPAM-PU/CMC@GEM + NIR group compared to PNIPAM-PU/CMC@GEM. The findings indicated that, PNIPAM-PU/CMC@GEM + NIR induced hydrogel increases ROS generation in KYSE-140 cells. Hydrogel plays a pivotal role in ROS production, whereas GEM enhances oxidative stress. Elevated ROS levels in KYSE-140 cells indicate the efficacy of hydrogel + NIR treatment. Thus, PNIPAM-PU/CMC@GEM + NIR enhances ROS production to trigger apoptosis, presenting a promising approach for the treatment of ESCC, particularly in resistant types [53].

### Wound scratch assay

**Fig. 8:**
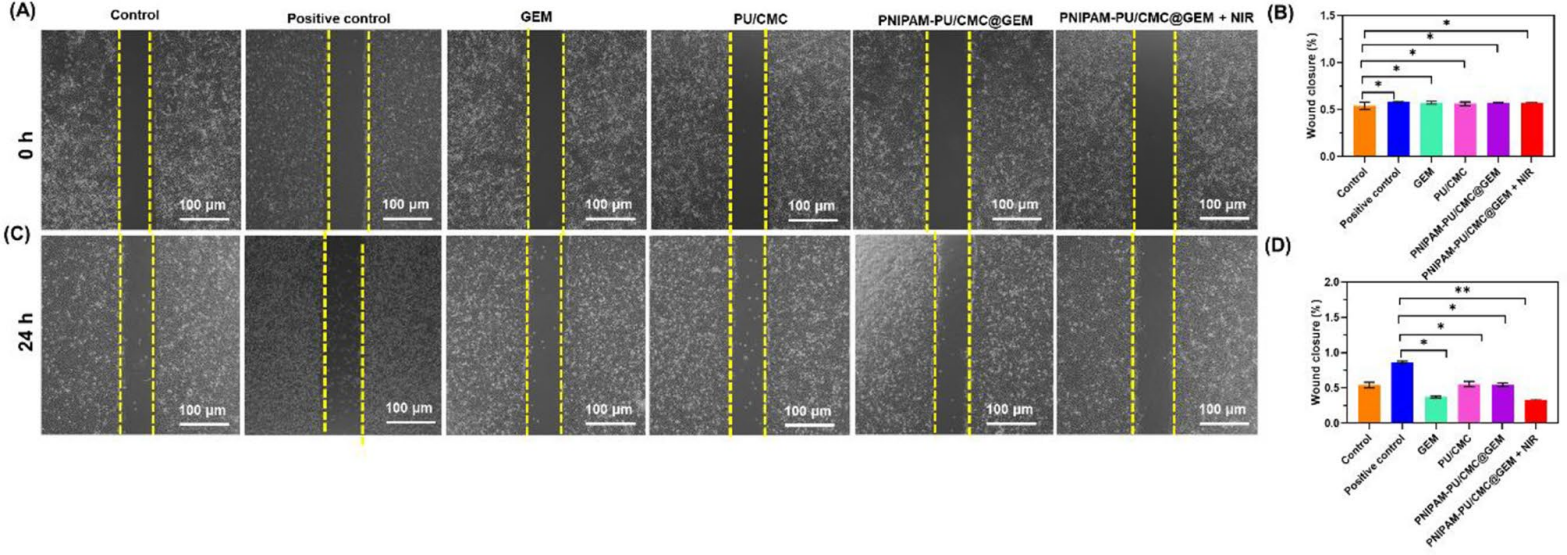
(**A** & **C**) Effects of VEGF (positive control), GEM, PU/CMC, PNIPAM-PU/CMC@GEM and PNIPAM-PU/CMC@GEM + NIR on in vitro scratch assays of KYSE-140 cells in 0 h and 24 h exposure; (**B** & **D**) Quantitative analysis of cell migration in wound scratch assay after treated with VEGF (positive control), GEM, PU/CMC, PNIPAM-PU/CMC@GEM and PNIPAM-PU/CMC@GEM + NIR in 0 h, and 24 h. Data were presented as mean ± SD (*n* = 3, *p < 0.05)

Figure 8 A & B illustrates the results of the wound healing assay conducted on KYSE-140 cells after treated with EGF (positive control), GEM, PU/CMC, PNIPAM-PU/CMC@GEM, and PNIPAM-PU/CMC@GEM + NIR for 0 h and 24 h. Figure 8B & D reveals the quantitative analysis wound closure in KYSE-140 cells. The findings displayed distinct differences in treatment groups. Untreated control cells exhibited substantial wound closure after 24 h indicating high migratory capacity. Treatment with PNIPAM-PU/CMC@GEM modestly inhibited migration, while PNIPAM-PU/CMC@GEM + NIR significantly reduced wound closure. Notably, PNIPAM-PU/CMC@GEM + NIR demonstrated the most pronounced inhibition of migration in KYSE-140 cells, with minimal wound closure observed after 24 h. The results displayed that PNIPAM-PU/CMC@GEM + NIR induced hydrogel considerably suppress cell migration, likely through a combination of cytotoxic effects, elevated oxidative stress, and apoptosis. Further, EGF positive control demonstrated significantly increased wound closure compared to the untreated cells, thereby validating our assay conditions. Inclusion of this migration promoting control allowed a more robust comparison, clearly highlighting the anti-migratory effect of the PNIPAM-PU/CMC@GEM + NIR hydrogel system. Images of the wound area were captured at 0 h and 24 h post treatment, and the gap closure was quantified using ImageJ software.

The enhanced inhibition after treated with GEM, PU/CMC, PNIPM-PU/CMC@GEM, and particularly the PNIPAM-PU/CMC@GEM + NIR groups underscore the role PU/CMC in ROS generation which leads cell apoptosis. The superior efficacy of PNIPAM-PU/CMC@GEM + NIR compared to PNIPAM-PU/CMC@GEM highlights the significance of NIR activation in enhancing apoptosis responses.

### Flowcytometry analysis

**Fig. 9:**
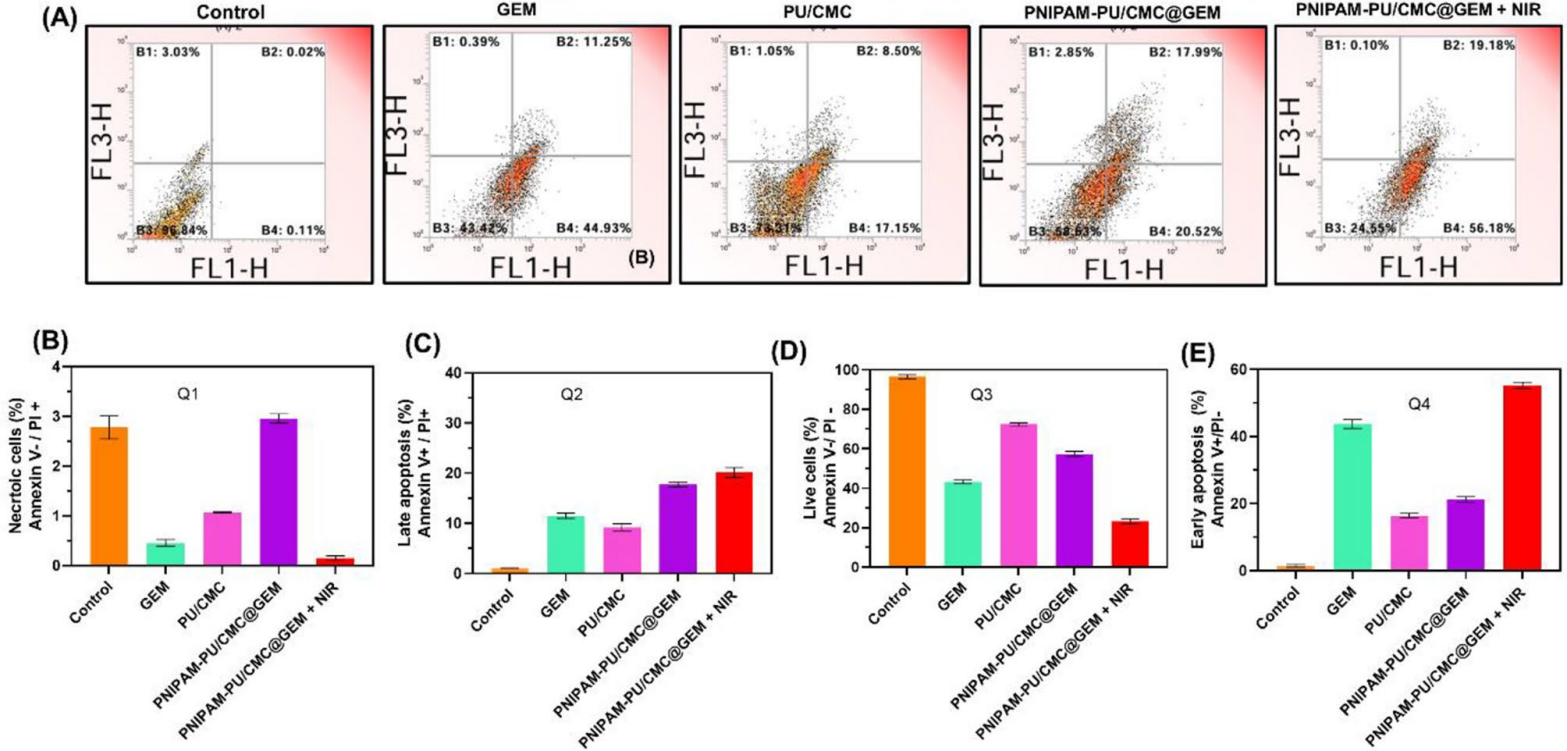
(**A**) Apoptosis detection using Annexin V-FITC/PI was performed using flow cytometry on A549 cells after treated with GEM, PU/CMC, PNIPAM-PU/CMC@GEM, and PNIPAM-PU/CMC@GEM + NIR; (**B-D)** The quantification of early apoptosis, late apoptosis, necrotic and live cells of different groups based on the flow cytometry results. Data are expressed as mean ± SD, *n* = 5. *p* ≤ 0.05

Flowcytometry analysis was used to quantify the cell apoptosis. The flow cytometry-based apoptosis detection and quantification of KYSE-140 cell apoptosis levels are presented in Fig. 9 A. Quantification of apoptosis levels including live cells, necrotic cells, early apoptotic cells and late apoptotic cells of KYSE-140 cells after treated with GEM, PU/CMC, PNIPAM-PU/CMC@GEM, and PNIPAM-PU/CMC@GEM + NIR were detected by flowcytometry and shown in Fig. 9B-E. The results displayed that, the cells in untreated group were viable, with low apoptosis rates according to flowcytometry with Annexin V-FITC/PI staining. Early apoptotic cells were marginally elevated after treatment with PNIPAM-PU/CMC@GEM hydrogel suggests low levels of cytotoxicity. However, when infused with NIR, maximum apoptosis was noticed which evidences the increased apoptosis via ROS production and DNA damage, in PNIPAM-PU/CMC@GEM + NIR hydrogel. With a significant increase in both early and late apoptotic cells, the PNIPAM-PU/CMC@GEM + NIR showed the most significant effects, suggesting that they successfully induced necrosis and apoptosis. The results indicate that the synergistic effects of GEM which intercalates into DNA and disrupts replication, PNIPAM-PU/CMC generate ROS and induce oxidative stress in the presence of NIR suggest that the combination of PNIPAM-PU/CMC and GEM with NIR considerably increases cytotoxicity and triggers programmed cell death through multiple mechanisms. Furthermore, a significant shift toward late-stage apoptosis and necrosis was observed in NIR irradiated group which showed an even more pronounced increase in apoptosis compared to PNIPAM-PU/CMC@GEM group. The NIR treated group showed increased apoptosis, demonstrating the importance of thermoresponsive and NIR responsive characteristics of hydrogel [54]. To maximize drug delivery and enhance the therapeutic effect of the fabricated hydrogel with GEM at tumor site, PNIPAM’s thermoresponsive nature allows localized heating and drug release, while NIR provide fine spatial control. These characteristics provide an effective strategy for enhancing the efficiency of cancer treatments by reducing systemic toxicity and increasing therapeutic outcomes by the induction of apoptosis through several mechanisms.

### In vivo studies

#### qRT-PCR analysis

**Fig. 10:**
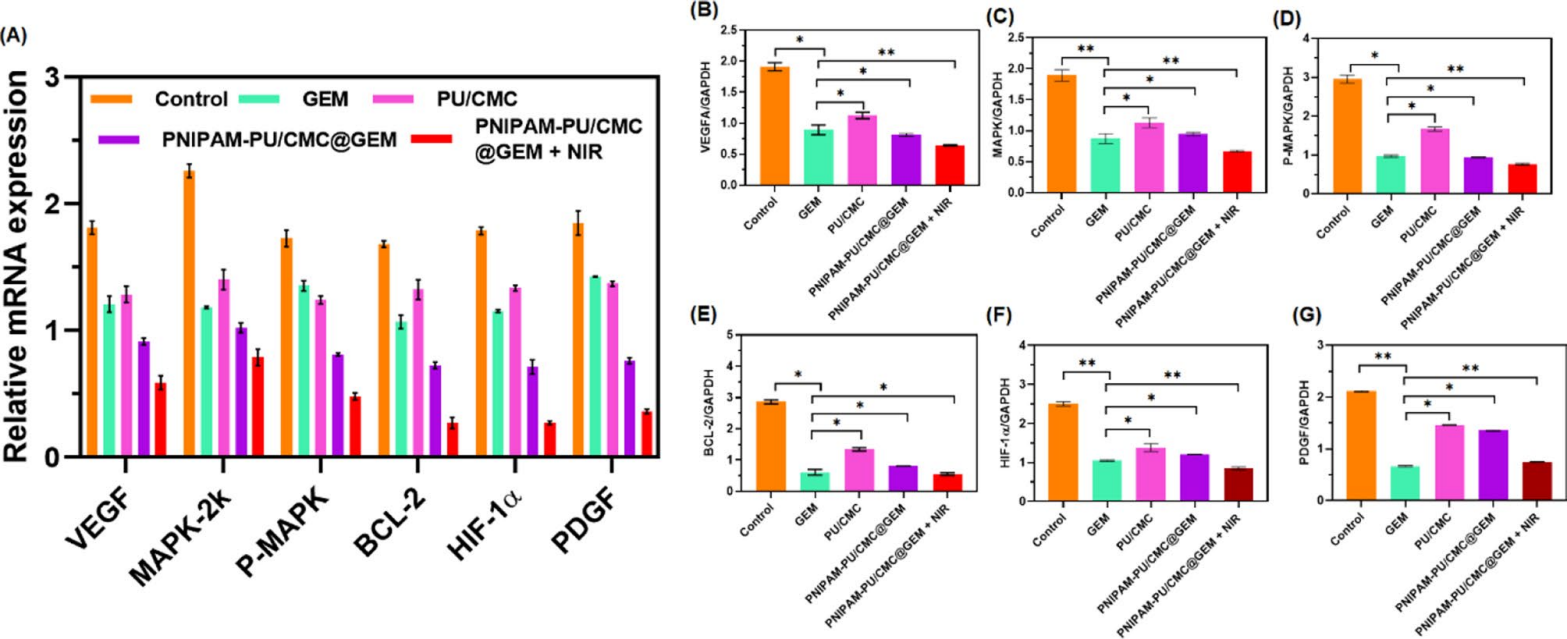
Effect of GEM, PU/CMC, PNIPAM-PU/CMC@GEM and PNIPAM-PU/CMC@GEM + NIR on (**A**) VEGFA, (**B**) MAPK, (**C**) P-MAPK, (**D**) BCL-2, (**E**) HIF-1α, and (**F**) PDGF pathways analysed by Western blot analysis. (**F**) The impact of GEM, PU/CMC, PNIPAM-PU/CMC@GEM and PNIPAM-PU/CMC@GEM + NIR on mRNA gene expression was determined by qRT-PCR analysis. Data are expressed as mean ± SD, *n* = 5. *p* ≤ 0.05

Figure 10 A, denotes the quantitative RT-PCR analysis of pro-apoptotic and anti-apoptotic signaling genes such as MAPK, VEGFA, BCL-2, P-MAPK, HIF-1α and PDGF in tumor cells after treated with GEM, PU/CMC, PNIPAM-PU/CMC@GEM and PNIPAM-PU/CMC@GEM + NIR. The findings displayed that, the expression of MAPK, VEGFA, BCL-2, P-MAPK, HIF-1α and PDGF gens was considerably increased in GEM, PU/CMC, and PNIPAM-PU/CMC@GEM treated groups (*P* < 0.05). Significantly, following the downregulation of VEGFA, the expression levels of MAPK, P-MAPK, BCL-2, HIF-1α and PDGF demonstrated a marked decrease, indicating a regulatory mechanism involving VEGFA. The findings demonstrated that, VEFGA plays a crucial role in regulating MAPK signaling and apoptotic pathways inside the tumour microenvironment. Moreover, results from the Western blog analysis corroborated the expression of MAPK, P-MAPK, BCL-2, HIF-1α and PDGF across all groups (Fig. 10B-G). A notable decrease in MAPK, P-MAPK, and BCL-2 protein levels was detected following VEGFA inhibition. The inhibition of VEFGA results in the reduction of MAPK phosphorylation and the downregulation of the anti-apoptotic marker BCL-2, indicating that targeting VEGFA may represent a viable therapeutic option in the context of cancer. These results are in line with previous research that has inhibiting cancer cell survival and increasing apoptosis can be achieved by targeting VEGFA [55]. Combining GEM, PNIPAM-PU/CMC and NIR exposure amplifies pro-apoptotic signaling, which may be driven by the synergistic effects. These results suggest that VEGFA could be a potential therapeutic target for enhancing the effectiveness of cancer treatments [56]. Further, the expression levels of HIF-1α and PDGF were assessed in tumor tissues harvested from each treatment group. The findings displayed that, the PNIPAM-PU/CMC@GEM + NIR treatment group exhibited a marked downregulation of both HIF-1α and PDGF in comparison to control and other treatment groups, further supporting the anti-angiogenic potential of the NIR therapy. The results support the observed VEGF downregulation and collectively confirm the mechanism of angiogenesis induced by PNIPAM-PU/CMC@GEM + NIR hydrogel. These results align with previous studies indicating that targeting VEGFA and related angiogenic pathways can effectively modulates VEGFA and its downstream effectors, presenting a promising strategy for enhancing chemotherapeutic efficacy.

#### Antitumor therapy

**Fig. 11:**
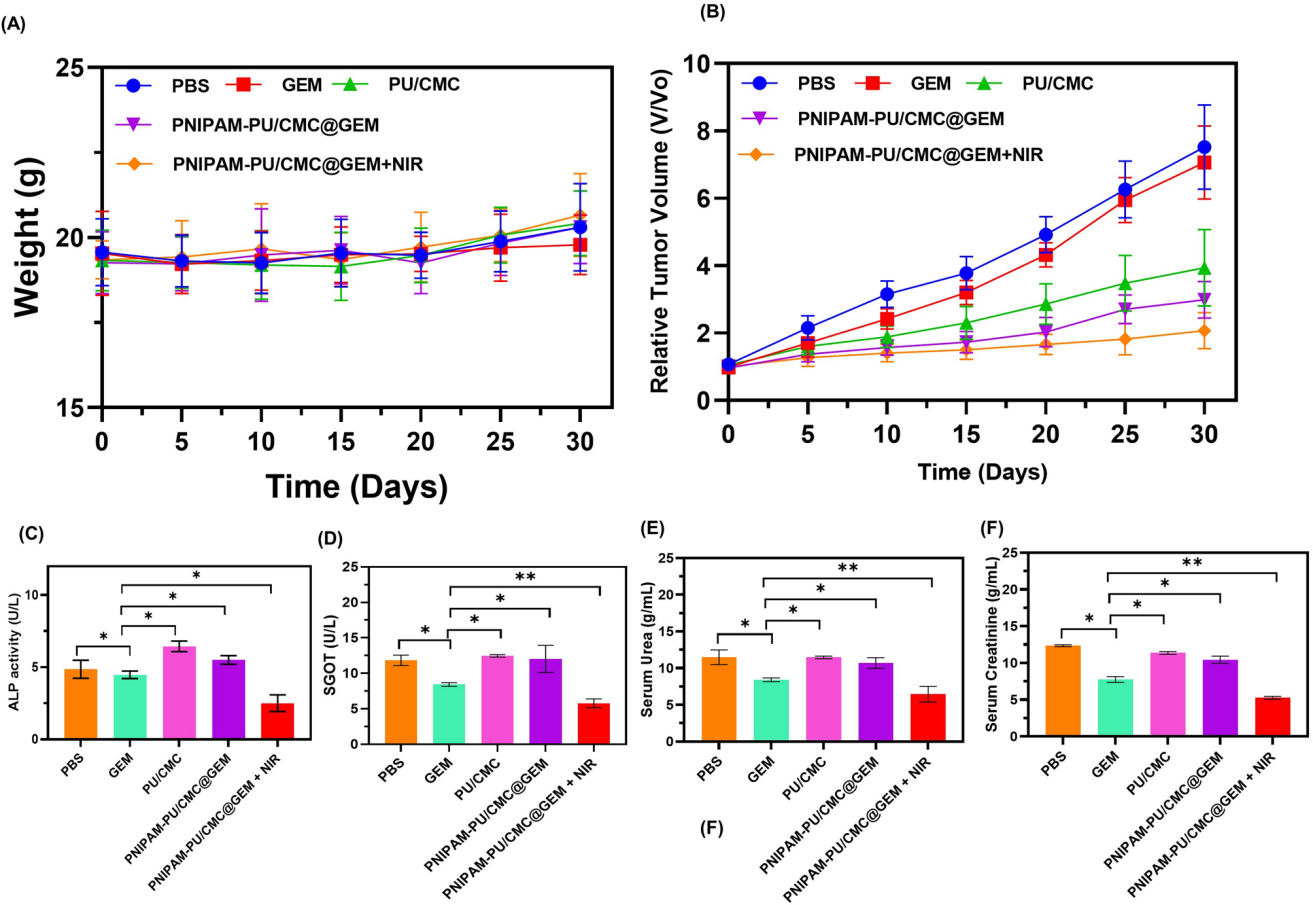
In vivo tumor biomarker analysis: (**A**) Body weight of the animal after tumor induction; (**B**) Tumor growth and tumor weight after treated with GEM, PU/CMC, PNIPAM-PU/CMC@GEM, and PNIPAM-PU/CMC@GEM + NIR; Quantification test on (**C**) ALP, (**D**) SGOT, (**E**) serum urea and (**F**) serum creatinine was analysed with after treated with GEM, PU/CMC, PNIPAM-PU/CMC@GEM, and PNIPAM-PU/CMC@GEM + NIR. Data are expressed as mean ± SD, *n* = 5. *p* ≤ 0.05

The current investigation assessed the efficacy and safety of a hydrogel treatment in a tumor bearing mice model that combined the thermoresponsive hydrogel PNIPAM-PU/CMC@GEM with NIR radiation. To assess the effect of GEM, PU/CMC, PNIPAM-PU/CMC and PNIPAM-PU/CMC + NIR, the weight of the mice model was evaluated after treatment. As shown in Fig. 11 A, all groups of animals, with the exception of the NIR treated group, gained weight. Over the course of 30 days experiment, the volume of the tumor was measured at alternate days with an electronic caliper, and the development of the tumour was monitored. The results showed that, animals maintained in Group I, the control group had a constant increase in tumor volume and a substantial increase in tumor growth (*P* < 0.05). Comparing the control group to animals treated with GEM, PU/CMC and PNIPAM-PU/CMC@GEM, it was shown that the tumor size was moderately reduced. However, significant reduction in tumor growth was observed in animals maintained in Group V which received PNIPAM-PU/CMC@GEM + NIR treatment as compared to group II, III and IV (Fig. 11B). This displays that, the exposure of KYSE-140 cells to GEM, PU/CMC and PNIPAM-PU/CMC@GEM did not cause significant systemic toxicity as evidenced by the increased tumor growth in these group animals. Conversely, the NIR induced group animals shows reduced tumor weight indicating a potential effect on tumor progression, necessitating further investigation into its application in animal physiology. Thus, the combined therapy such as NIR, thermoresponsive hydrogel and drug considerably improved the therapeutic efficacy. Combination therapy certainly has a synergistic impact, as shown by the tumor growth ratio. Notably, animals in PNIPAM-PU/CMC@GEM + NIR treated group did not have any weight loss compared to other groups. Furthermore, the results of the in vivo toxicity study reveal that PNIPAM-PU/CMC@GEM + NIR hydrogel did not exhibit any toxicity, as indicated by the levels of SGOT, SGPT, ALP, urea, and creatinine. Hepatic stress linked to tumour progression was shown by significantly higher level of ALP, SGOT, serum, urea and creatinine in tumor bearing group (Fig. 11 C-F). These enzyme levels were found to be significantly lower in the group treated with PNIPAM-PU/CMC@GEM + NIR compared to other groups. These results indicate that, the PNIPAM-PU/CMC@GEM with NIR group has strong anticancer properties with low toxicity.

#### Histopathology analysis

**Fig. 12:**
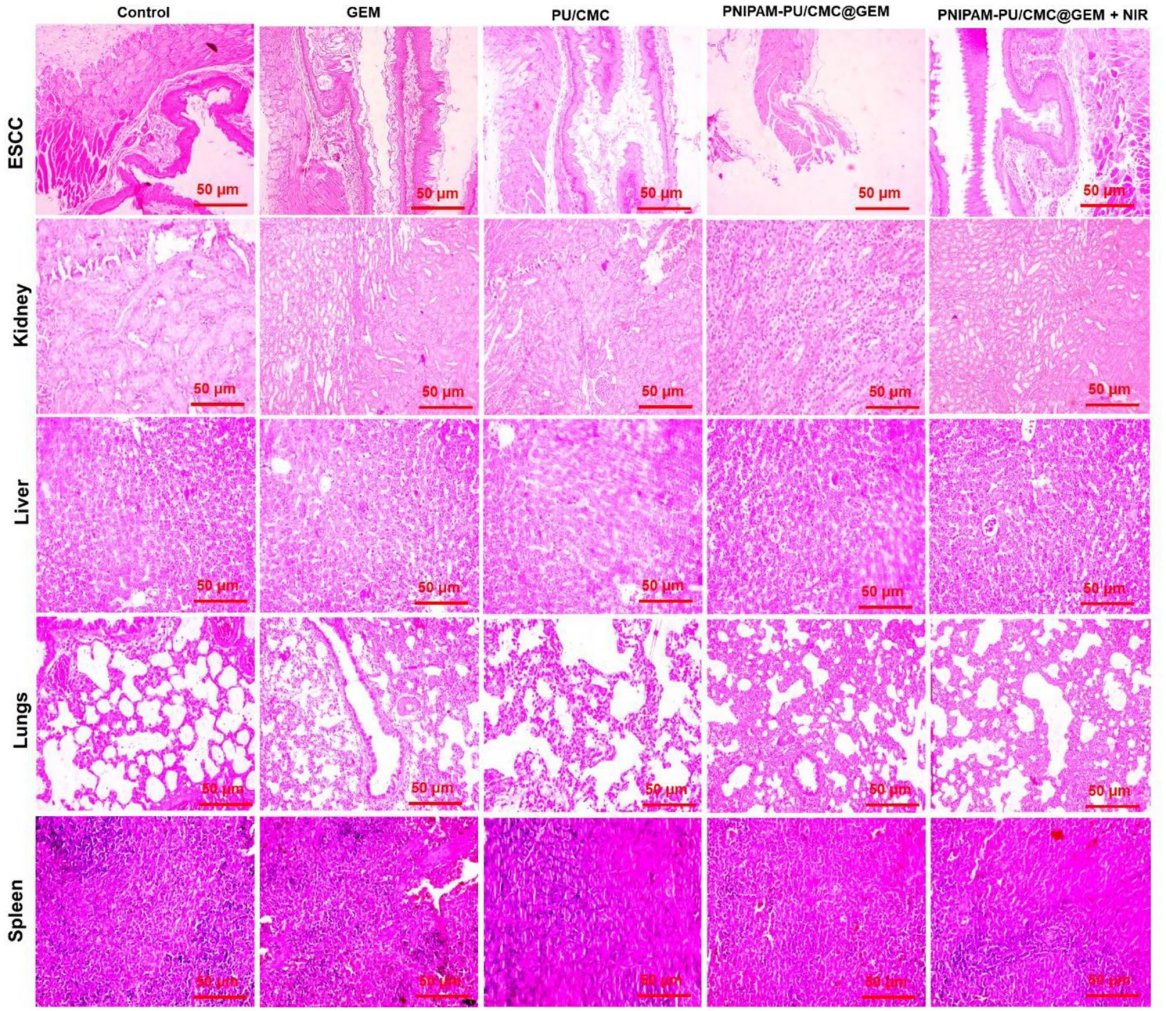
H&E-stained tissue sections of ESCC, kidney, liver, lungs, and spleen following exposure to GEM, PU/CMC, PNIPAM-PU/CMC@GEM and PNIPAM-PU/CMC@GEM + NIR

To assess the potential effect and toxicity of hydrogel treatment with GEM, PU/CMC, PNIPAM-PU/CMC@GEM and PNIPAM-PU/CMC@GEM + NIR, histopathological evaluations were performed on tissues derived from different organs. After being removed from the animal, the various organs including the spleen, kidney, liver, lungs and ESCC were stained using H & E staining and the sections were analysed under fluorescence microscopy. These findings suggest that, the NIR induced PNIPAM-PU/CMC@GEM hydrogel reduces the toxicity of chemotherapy to several organs, including kidneys, liver, lungs, and spleen, while exhibiting relatively low levels of damage when compared to other formulations. Tumor cell necrosis, as measured by morphological indicators such cell nuclear shrinkage, fragmentation, and nuclear condensation, varies in degree among animal tissues of different organs after exposure to manufactured hydrogel. Nevertheless, the PNIPAM-PU/CMC@GEM + NIR treated group showed most extensive areas of necrosis, suggesting that the tumor suppression was happened most successful (Fig. 12). Significant tumor necrosis and apoptosis are induced by PNIPAM-PU/CMC@GEM + NIR therapy, while normal tissue integrity is preserved. Thus, the fabricated hydrogel loaded with drug enhances cancer cell death by disrupting mitochondrial function in tumor cells, without generating damage to other organs. The results show that the combination of PNIPAM-PU/CMC@GEM hydrogel with NIR activation has great therapeutic potential for ESCC.

## Conclusion

In the present study, PNIPAM-PU/CMC@GEM hydrogel system was developed and evaluated for targeted drug delivery of GEM in the treatment of ESCC. The PNIPAM-PU/CMC hydrogel exhibited excellent physicochemical properties, including structural stability, temperature responsive behaviour and efficient drug encapsulation. In vitro assays reveals that the NIR induced PNIPAM-PU/CMC@GEM hydrogel significantly suppressed the viability, proliferation, and migration of KYSE-140 cells. Further, the JC-1 staining revealed a pronounced loss of mitochondrial membrane potential in treated cells, indicating apoptosis induction, particularly in the NIR triggered PNIPAM-PU/CMC@GEM group. In addition, H & E staining in in vivo tumor models showed a notable reduction in tumor growth and cell aggregation. The therapeutic efficacy was further supported by Western blot analysis, which reveals the downregulation of critical oncogenic markers, including MAPK, VEGFA, BCL-2, phosphorylated MAPK, HIF-1a, and PDGF. Collectively these findings highlight the promising effect of PNIPAM-PU/CMC@GEM as a biodegradable, thermoresponsive and NIR responsive drug delivery system for effective ESCC therapy with strong translational potential. Despite its promising efficacy, limitations such as shallow NIR penetration, potential GEM thermal degradation, and long-term PU-associated fibrosis must be addressed. Future studies should focus on optimizing laser parameters, enhancing biocompatibility, and evaluating performance in larger tumors.

## Electronic supplementary material

Below is the link to the electronic supplementary material.

Supplementary Material 1

## Data Availability

No datasets were generated or analysed during the current study.
