# Supplementary material for: A novel PNIPAM-Modified polyurethane/carboxymethyl cellulose photo-thermoresponsive hydrogel loaded with gemcitabine to suppress esophageal cancer cells via VEGF-mediated angiogenic pathway inhibition
Source: J Biol Eng. 2025 Jul 23;19:66. doi: 10.1186/s13036-025-00530-y (PMC12285038; doi:10.1186/s13036-025-00530-y)
Supplement: Supplementary file 1 — Supplementary Material 1 [file 13036_2025_530_MOESM1_ESM.docx]

**Supplementary figure. 1**


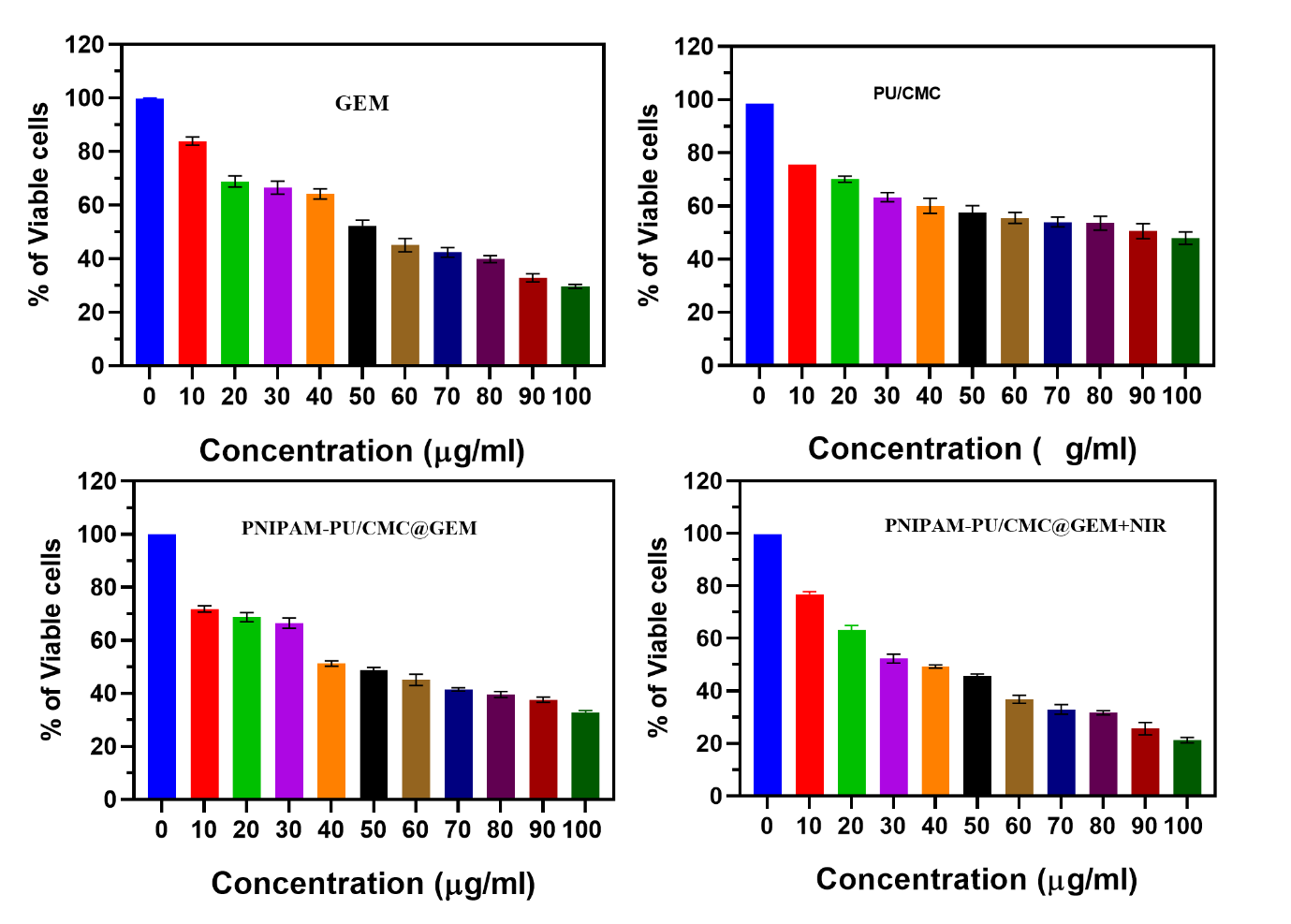


**Supplementary figure. 1.** IC_50_ concentration of GEM, PU/CMC, PNIPAM-PU/CMC@GEM and PNIPAM-PU/CMC@GEM+ NIR on the viability of KYSE-140 cells. Data were presented as mean ± SD (n = 3, **p <0.05*).
